# Distinct Modes of Regulation by Chromatin Encoded through Nucleosome Positioning Signals

**DOI:** 10.1371/journal.pcbi.1000216

**Published:** 2008-11-07

**Authors:** Yair Field, Noam Kaplan, Yvonne Fondufe-Mittendorf, Irene K. Moore, Eilon Sharon, Yaniv Lubling, Jonathan Widom, Eran Segal

**Affiliations:** 1 Department of Computer Science and Applied Mathematics, Weizmann Institute of Science, Rehovot, Israel; 2 Department of Biochemistry, Molecular Biology, and Cell Biology, Northwestern University, Evanston, Illinois, United States of America; 3 Department of Molecular Cell Biology, Weizmann Institute of Science, Rehovot, Israel; Duke University, United States of America

## Abstract

The detailed positions of nucleosomes profoundly impact gene regulation and are partly encoded by the genomic DNA sequence. However, less is known about the functional consequences of this encoding. Here, we address this question using a genome-wide map of ∼380,000 yeast nucleosomes that we sequenced in their entirety. Utilizing the high resolution of our map, we refine our understanding of how nucleosome organizations are encoded by the DNA sequence and demonstrate that the genomic sequence is highly predictive of the in vivo nucleosome organization, even across new nucleosome-bound sequences that we isolated from fly and human. We find that Poly(dA:dT) tracts are an important component of these nucleosome positioning signals and that their nucleosome-disfavoring action results in large nucleosome depletion over them and over their flanking regions and enhances the accessibility of transcription factors to their cognate sites. Our results suggest that the yeast genome may utilize these nucleosome positioning signals to regulate gene expression with different transcriptional noise and activation kinetics and DNA replication with different origin efficiency. These distinct functions may be achieved by encoding both relatively closed (nucleosome-covered) chromatin organizations over some factor binding sites, where factors must compete with nucleosomes for DNA access, and relatively open (nucleosome-depleted) organizations over other factor sites, where factors bind without competition.

## Author Summary

The detailed positions of nucleosomes along genomes have critical roles in transcriptional regulation. Consequently, it is important to understand the principles that govern the organization of nucleosomes in vivo and the functional consequences of this organization. Here we report on progress in identifying the functional consequences of nucleosome organization, in understanding the way in which nucleosome organization is encoded in the DNA, and in linking the two, by suggesting that distinct transcriptional behaviors are encoded through the genome's intrinsic nucleosome organization. Our results thus provide insight on the broader question of understanding how transcriptional programs are encoded in the DNA sequence. These new insights were enabled by individually sequencing ∼380,000 nucleosomes from yeast in their entirety. Using this map, we refine our previous model for predicting nucleosome positions and demonstrate that our new model predicts nucleosome organizations in yeast with high accuracy and that its nucleosome positioning signals are predictive across eukaryotes. We show that the yeast genome may utilize these nucleosome positioning signals to encode regions with both relatively open (nucleosome-depleted) chromatin organizations and relatively closed (nucleosome-covered) chromatin organizations and that this encoding can partly explain aspects of transcription factor binding, gene expression, transcriptional noise, and DNA replication.

## Introduction

DNA in eukaryotes is highly packaged into nucleosome arrays, which together compact ∼75–90% of the genome [1]. Because most DNA is wrapped in nucleosomes, and nucleosomes occlude their DNA from access to most other DNA binding proteins, revealing the detailed organization of nucleosomes across genomes and understanding the mechanisms that control their positioning is critical for understanding transcription factor binding and thus transcriptional regulation.

Several studies predicted in vivo nucleosome positions directly from the DNA sequence [2]–[6], suggesting that nucleosome organizations are partly encoded in the genomic sequence itself, through the nucleosomes' intrinsic DNA sequence preferences, which vary greatly between differing DNA sequences [7],[8]. However, an intriguing and less explored question concerns the functional roles that this encoding may have.

Studying this question requires detailed measurements of nucleosome organizations and availability of large-scale functional genomic data with which to compare these measurements. We thus focused on yeast, where many dynamic aspects of transcriptional regulation have been experimentally measured genome-wide, and where nucleosome occupancy have been measured using DNA microarrays [5],[9],[10]. To improve the resolution of the measured nucleosome organization, we used a parallel sequencing technology whose reads are longer than one nucleosome length, and obtained ∼380,000 fully sequenced yeast nucleosomes, resulting in a genome-wide map of nucleosome occupancy with high accuracy and dynamic range. While this manuscript was in review, two other studies that used parallel sequencing to map nucleosomes were published [11],[12].

Here, we first use our map to better understand how nucleosome organizations are encoded by intrinsic signals in genomic DNA, and find that the genomic sequence is highly predictive of nucleosome organizations in yeast. By isolating nucleosome-bound sequences from fly and human, we further show that the key positioning signals in yeast are also predictive of nucleosome organizations in higher eukaryotes. Our results suggest that the yeast genome utilizes these intrinsic nucleosome positioning signals to encode both relatively open (nucleosome-depleted) and relatively closed (nucleosome-covered) chromatin organizations, resulting in two distinct modes of regulation by chromatin with different biological functions. In promoters that encode relatively open chromatin architectures, transcription factors can access their sites more freely, resulting in a homogeneous cell population with relatively low cell-to-cell expression variability, or transcriptional noise. Genes associated with these promoters are enriched in essential genes and in ribosomal protein genes. In contrast, in promoters that encode relatively closed chromatin architectures, factors compete with nucleosomes for access to the DNA, resulting in a heterogeneous cell population with higher transcriptional noise. Genes associated with these promoters are enriched in non-essential genes and in genes that are active only in specific biological conditions. Finally, we provide evidence that the encoding of relatively open and closed chromatin architectures may also play a role in DNA replication, such that replication origins that encode open chromatin organizations initiate replication with higher efficiency. Taken together, our results reveal new insights into the mechanisms by which the genomic DNA sequence dictates the nucleosome organization, and by which genomically encoded nucleosome organizations may influence chromosome functions.

## Results

### Obtaining a Single Molecule Map of Nucleosome Positions

To obtain a single molecule map of yeast nucleosomes, we isolated mononucleosomes from eight independent biological replicates and fully sequenced ∼503,000 of the nucleosome DNAs, using a parallel sequencing technology whose sequence reads are ∼200 bp long. Thus, aside from the limitations imposed by using micrococcal nuclease to isolate nucleosomes, our approach is optimal for mapping nucleosomes, since it extracts only the DNA segments of interest with little flanking DNA, and then reads them in full. Such full length nucleosome reads allow us to map the nucleosome organization with potentially greater resolution compared to approaches that map only one nucleosome end, because the kinetics of nuclease digestion result in nucleosomal DNA fragments that vary in length relative to the canonical 147 bp nucleosome, and thus, mapping only one end leaves considerable uncertainty regarding the location of the other end, for any given nucleosome DNA molecule. In addition, the sequencing method affords a large dynamic range, limited only by the number of sequence reads obtained. Compared to using microarrays as the readout of nucleosome occupancy, a sequencing-based approach provides an experimental decomposition of the average nucleosome occupancy, such as that measured by microarrays, into discrete nucleosome configurations.

**Figure 1:**
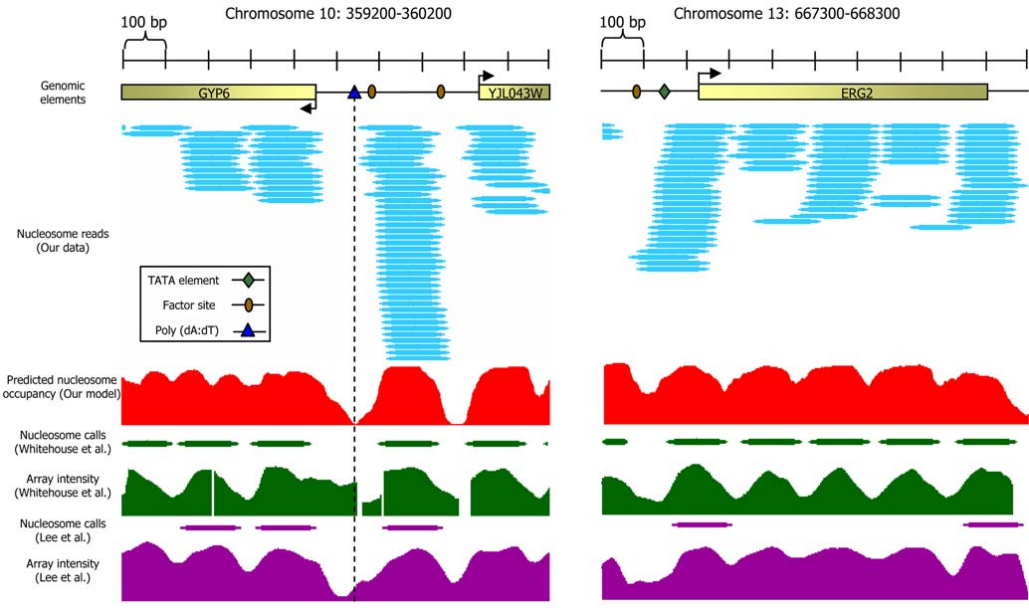
Nucleosome organization at two genomic regions. Shown are the raw data measured in this study at two 1000bp-long genomic regions. Every cyan oval represents the genomic location of one nucleosome that we sequenced in its entirety. Also shown is the average nucleosome occupancy per basepair predicted by the sequence-based nucleosome model that we developed here (red), the raw hybridization signal of two microarray-based nucleosome maps [5],[10] (green and purple traces), and the locations of nucleosomes that were computationally inferred from these hybridization signals [5],[10] (green and purple ovals). Note that although the nucleosome calls from the microarray maps are close to nucleosome locations from our map, the microarray map does not reveal the underlying variability in the detailed nucleosome read locations that we observe in our data. Annotated genes [63], transcription factor binding sites [47], TATA sequences [53], and Poly(dA:dT) elements in the region are also shown (top).

After excluding nucleosomes that map to repetitive regions, we obtained ∼380,000 uniquely mapped nucleosomes such that on average, every basepair is covered by five nucleosome reads (Figure 1). To validate our nucleosome map, we compared it to ∼100 nucleosome positions mapped using conventional sequencing [2], three large collections of generic nucleosomes mapped using microarrays [5],[9],[10], and two collections of generic [11] and H2A.Z [13] nucleosomes mapped by sequencing one end of each nucleosome. Our map shows significant correspondence with all existing maps but differs in both the detailed locations and occupancy of many measured nucleosomes (Figure S1).

### The Genomic Sequence Is Highly Predictive of Nucleosome Occupancy

Before exploring the functional consequences of the intrinsically encoded nucleosome organization, we used the high resolution of the sequence-based nucleosome map to refine our understanding of how nucleosome organizations are encoded by the genomic sequence. Several models for predicting nucleosome positions from DNA sequence were recently constructed [2]–[6]. Our motivation for constructing a new model was twofold. First, none of these models were constructed from a genome-wide map of nucleosome positions based on direct sequencing, and we thus sought to utilize the high resolution and accuracy of such a map for constructing a model. Our second motivation was to combine into one model, two primary components that were each, separately, the basis of the previously published models. One of these components consists of periodicities of specific dinucleotides along the nucleosome length, on which earlier models were based [2],[3]. The other component includes sequences that are generally disfavored by nucleosomes, regardless of their position along the nucleosome length, whose incorporation was shown to increase the predictive power [4]–[6].

**Figure 2:**
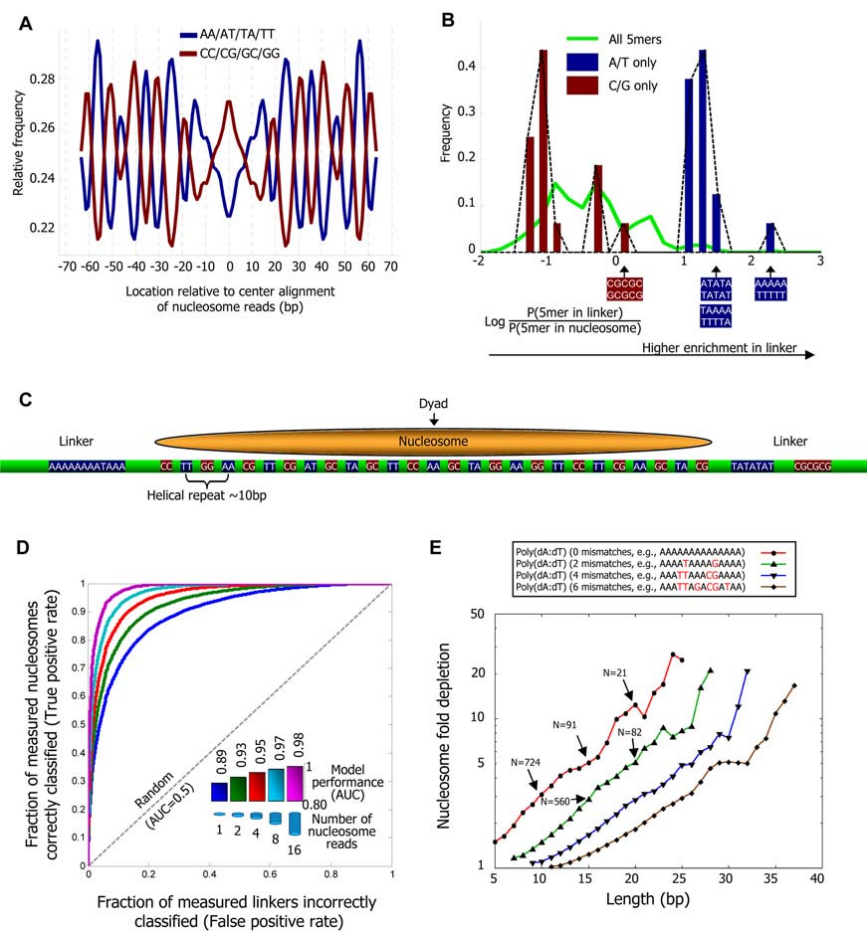
Nucleosome positioning signals in genomic sequence. (A) Fraction (normalized, see Methods) of AA/AT/TA/TT and separately, CC/CG/GC/GG dinucleotides at each position of our center-aligned nucleosome-bound sequences with length 146–148, showing ∼10 bp periodicity of these dinucleotide sets. (B) Many 5-mers are enriched in linker or nucleosome regions. Shown is the distribution of (log base 2) ratios between the frequency of 5-mers in linker regions and in nucleosomal DNA regions for all 5-mers (green line), and for the 32 5-mers composed exclusively of either G/C (red bars) or A/T (blue bars) nucleotides. Linkers are taken as contiguous non-repetitive regions of lengths 50–500 bp that are not covered by any nucleosome read in our data. (C) Illustration of the key features of our probabilistic nucleosome–DNA interaction model, including the periodic dinucleotides patterns preferred within the nucleosome, and the 5-mers preferred in linkers. (D) Our model classifies linkers from nucleosomal DNA with high accuracy. Shown is the fraction of all measured nucleosomes that our model correctly classifies as nucleosomes (*y*-axis; true positive rate) against the fraction of all measured linkers that our model incorrectly classifies as nucleosomes (*x*-axis; false positive rate), for each possible threshold on the minimum score above which our model classifies a region as nucleosomal. The score of each measured nucleosome or linker is the mean score that our model assigns in the region that is within 20 bp from the center of the nucleosome or linker, respectively. Scores of the model are assigned using a cross validation scheme, in which every measured nucleosome or linker on a given chromosome is assigned a score using a model that was trained from the data of all other chromosomes. Linkers are defined as contiguous non-repetitive regions of lengths 50–500 bp that are not covered by any nucleosome in our data. Results are shown for separating these 8,017 linkers from nucleosomes with various levels of occupancy (1, 2, 4, 8, and 16), where the occupancy of a nucleosome is defined by the number of nucleosome reads whose center is within 20 bp of its own center. The number of nucleosomes in each classification group are 84,410 (occupancy 1), 69,703 (occupancy 2), 38,787 (occupancy 4), 12,076 (occupancy 8), and 1,601 (occupancy 16). (E) Shown is the combined nucleosome fold depletion over all homopolymeric tracts of A or T (Poly(dA:dT) elements) of length *k*, for *k* = 5,6,7,…, and for Poly(dA:dT) elements with exactly 0, 2, 4, or 6 base substitutions (mismatches). Each graph is trimmed at a length *K* in which there are less than 10 elements, and the fold depletion at this final point is computed over all elements whose length is at least *K*. The combined fold depletion of a set of genomic elements (*y*-axis) is the ratio between their expected and observed nucleosome coverage, where the expected coverage is the average coverage of any basepair according to our data, and the observed coverage is the average coverage of a basepair from the set (see Methods). The number of underlying elements at various points in the graph is indicated (*N*). See Figure S4 for a graph of all possible mismatches and showing the number of elements at all points.

**Figure 3:**
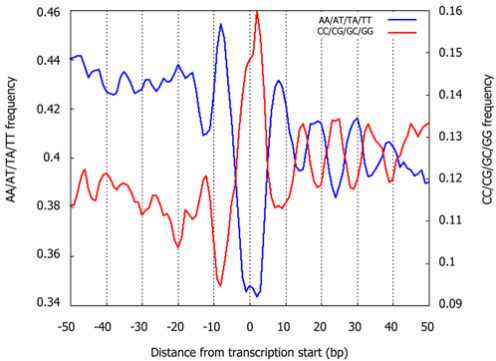
Periodicity of A/T and G/C dinucleotides around transcription start sites in yeast. Shown is the frequency of dinucleotides composed exclusively of T/A dinucleotides (blue line), or of G/C dinucleotides (red line) around transcription start sites of yeast genes. Both sets of dinucleotides exhibit ∼10 bp periodicities, but with opposite phases, across a ∼50 bp region.

Regarding the periodic component, several studies [2],[3],[14],[15] characterized the nucleosomes' intrinsic sequence preferences primarily by ∼10 bp periodicities of specific dinucleotides along the nucleosome length, thought to facilitate the sharp bending of DNA around the nucleosome [16]. We find similar periodicities in our new large nucleosome collection, demonstrating that these periodic dinucleotides are important genome-wide (Figure 2A and Figure S2). These same periodicities also arise in H2A.Z-containing nucleosomes [13], and in every in vivo and in vitro nucleosome collection obtained by direct sequencing from any organism [2], [11], [15], [17]–[19]. Moreover, these periodicities are also present in yeast transcription start sites (Figure 3), worm introns, 5′ and 3′ UTRs [20], human CpG dinucleotides not in CpG islands [21], and HIV integration sites in human [22].

Other studies [4]–[6] focused on sequences that are generally disfavored by nucleosomes, regardless of their detailed position along the nucleosome. We thus used our map to systematically identify sequences that are generally disfavored by nucleosomes, by extracting from our map contiguous regions not covered by any nucleosome, and comparing the frequencies of 5-mers in these linker DNA regions to their frequencies in the nucleosome-bound sequences. Indeed, we find that many 5-mers are enriched in linkers, including AAAAA as the most dominant signal, as well as all other 5-mers composed exclusively of A/T nucleotides, and the repetitive sequence CGCGC, shown to disfavor nucleosome formation [23] (Figure 2B). Notably, these same 5-mers are enriched in nucleosome-depleted regions from human [24], further suggesting that they represent nucleosome-disfavoring elements, and that such disfavoring elements may be universal.

We thus constructed a probabilistic nucleosome–DNA interaction model that integrates both the (nucleosome-favorable) position-specific periodic component and the (nucleosome-disfavoring) position-independent 5-mer component, and scores the nucleosome formation potential of every 147 bp sequence as the ratio between these components (Figure 2C). In our model, the periodic component dictates the high-resolution positioning of nucleosomes (known as the rotational setting), because its ∼10 bp periodicity results in strongly correlated scores between genomic positions separated by 10 bp, and strongly anti-correlated scores between positions separated by 5 bp. In contrast, the 5-mer nucleosome-disfavoring component scores each 147 bp sequence based on the set of its constituent 5-mers without regard to their exact position within the 147 bp sequence. Thus, scores of the 5-mer component primarily vary over longer genomic distances and hence this component dictates the absolute level of nucleosome occupancy of a region (known as the translational setting).

To validate our new model, we tested whether its predictions agree with the in vivo nucleosome map at the scale of individual nucleosomes. Specifically, we defined linkers as contiguous regions of lengths 50–500 bp that are not covered by any nucleosome, and evaluated the model's ability to separate these linkers from sets of nucleosomes with various levels of occupancy (1, 2, 4, 8, and 16), where the occupancy of a nucleosome is defined by the number of nucleosome reads whose center is within 20 bp of its own center. We then scored each of the resulting linkers and nucleosomes as the mean score (identical results were obtained by selecting the max score) that our model assigns to the region that is 20 bp from the center of the linker or nucleosome, respectively. We used a cross validation scheme, in which model predictions on any given chromosome are computed from a model whose parameters were estimated only from the data of all other chromosomes. This way we can generate genome-wide nucleosome occupancy predictions at each chromosome, where the predictions on each chromosome were computed from models that were trained on other chromosomes. We use these cross-validation predictions in all of the following validation analyses.

If the model were fully predictive of our in vivo map, then the model score of every nucleosomal region would be higher than that of every linker region. A standard quantification of this predictive power is the receiver operating characteristic (ROC) curve, whose area under the curve (AUC) is 1 for perfect performance and 0.5 for random guessing. We found a near-perfect AUC performance of 0.97 in separating ∼8,000 linkers from ∼12,000 regions that contain nucleosomes with a high occupancy of at least 8 nucleosome reads, and an AUC of 0.89 for separating these ∼8,000 linkers from ∼84,000 regions that contain nucleosomes with the minimal possible occupancy of one nucleosome read (Figure 2D). For example, at the model score threshold in which 90% (true positive rate) of the nucleosomes of occupancy 8 are correctly predicted, less than 10% (false positive rate) of the linkers are incorrectly predicted as nucleosomes. The absolute performance in these tests is remarkable, and demonstrates that our model is highly predictive of nucleosome occupancy in yeast. We also find that the performance of the model in this cross validation scheme is nearly identical to its performance on the training data, suggesting that our model does not overfit the input data (Figure S3). The fact that the model performs better in classifying nucleosomes with higher occupancy indicates that the probability that a nucleosome will occupy a region within the genome is higher at regions that match the sequence preferences of nucleosomes, as represented by our model. Note that since our predictions are done in a cross validation scheme, this result is not a trivial consequence of our training procedure, since a trained model does not have access to the level of occupancy of the nucleosomes on which its predictions are tested.

To calibrate the performance of our model, we compared it to the performance of previously published methods, and found that our model performs better than previous approaches when tested on our data (Figure S3). Similarly, we observed highly significant predictive power on two microarray-based nucleosome maps [5],[10] (Figure S3). Here, three models achieved the best, equivalent performance [5],[6], and our model was among them. Despite the outcome of these comparisons, we note that it is difficult to conclude from these tests which model is best, since for such an objective evaluation, each model should be trained using exactly the same input data, and such a comparison is out of our current scope and objective. Nevertheless, the performance of all of these models strongly supports the overall conclusion that the genomic sequence is highly predictive of nucleosome organizations in yeast.

**Figure 4:**
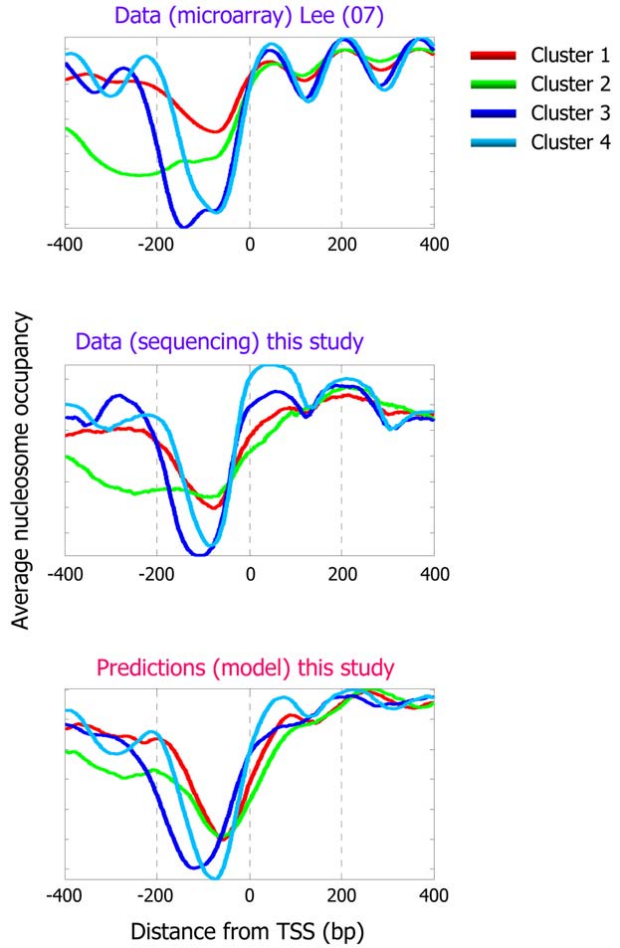
Our model predicts distinct nucleosome organizations around transcription start sites. Shown is the average nucleosome organization around transcription start sites of four sets of genes that were reported in [5] by clustering their measured nucleosome occupancy profiles. One of the four clusters reported in [5] corresponds to promoters that lack a significant nucleosome depleted region (cluster 1; red line in plots). The other three clusters have a clear nucleosome depleted region in their promoters, and are also reported in [5] as enriched for protein biosynthesis (cluster 2; green line), ribosome biogenesis (cluster 3; blue line), and protein modification (cluster 4; cyan line). The average nucleosome occupancy is shown from the original data of [5] (top) that was used for the clustering, and for our data (middle), as well as for the predicted occupancy of the nucleosome positioning model that we developed here (bottom).

Recent analyses of genome-wide nucleosome occupancy measurements in yeast identified different classes of nucleosome occupancy patterns in gene promoters, by clustering the nucleosome occupancy patterns [5]. Notably, we find that our model is also able to accurately predict the occupancy patterns of these different classes, suggesting that these differing nucleosome occupancy patterns are partly encoded in the DNA sequence, through the nucleosome sequence preferences (Figure 4).

Taken together, we conclude, in accord with other recent studies [2]–[6], that the genomic sequence is highly predictive of the nucleosome organization in yeast.

### Universal Genomic Signals for Nucleosome Positioning

Finally, we tested whether the nucleosome positioning signals of our model are also predictive of nucleosome occupancy in higher eukaryotes. To this end, we obtained nucleosome datasets from yeast [13], worm [17], and chicken [15], and also isolated and sequenced two new independent nucleosome collections from fly and two from human. Since there is variability in the base composition of different regions in the human genome, in one of the human collections, we extracted nucleosome-bound sequences from regions of the human genome that are strongly enriched in G/C nucleotides (60% G/C, see Methods), allowing us to evaluate the model performance on regions with atypical base compositions. In addition, we isolated and sequenced nucleosomes reconstituted in vitro on human genomic DNA and also obtained a previous such in vitro-selected collection from yeast [2], allowing us to test whether the model mainly captures nucleosome sequence preferences (since the in vitro experiments are done with purified histone octamers assembled on purified genomic DNA). To test whether the nucleosome positioning signals that we find in yeast are also important in these in vitro collections and in the collections from higher eukaryotes, we evaluated the model's performance locally around the ∼200–2000 nucleosomes that were mapped in each collection. The idea behind this test is that relative to the genomic location of a given nucleosome-bound sequence, a predictive model should assign higher scores to the position of that sequence, compared, for example, to scores that it assigns to positions that are half a nucleosome away from that position. For all of the following tests, we used our above model, learned only from the nucleosome-bound sequences that we measured in yeast.

**Figure 5:**
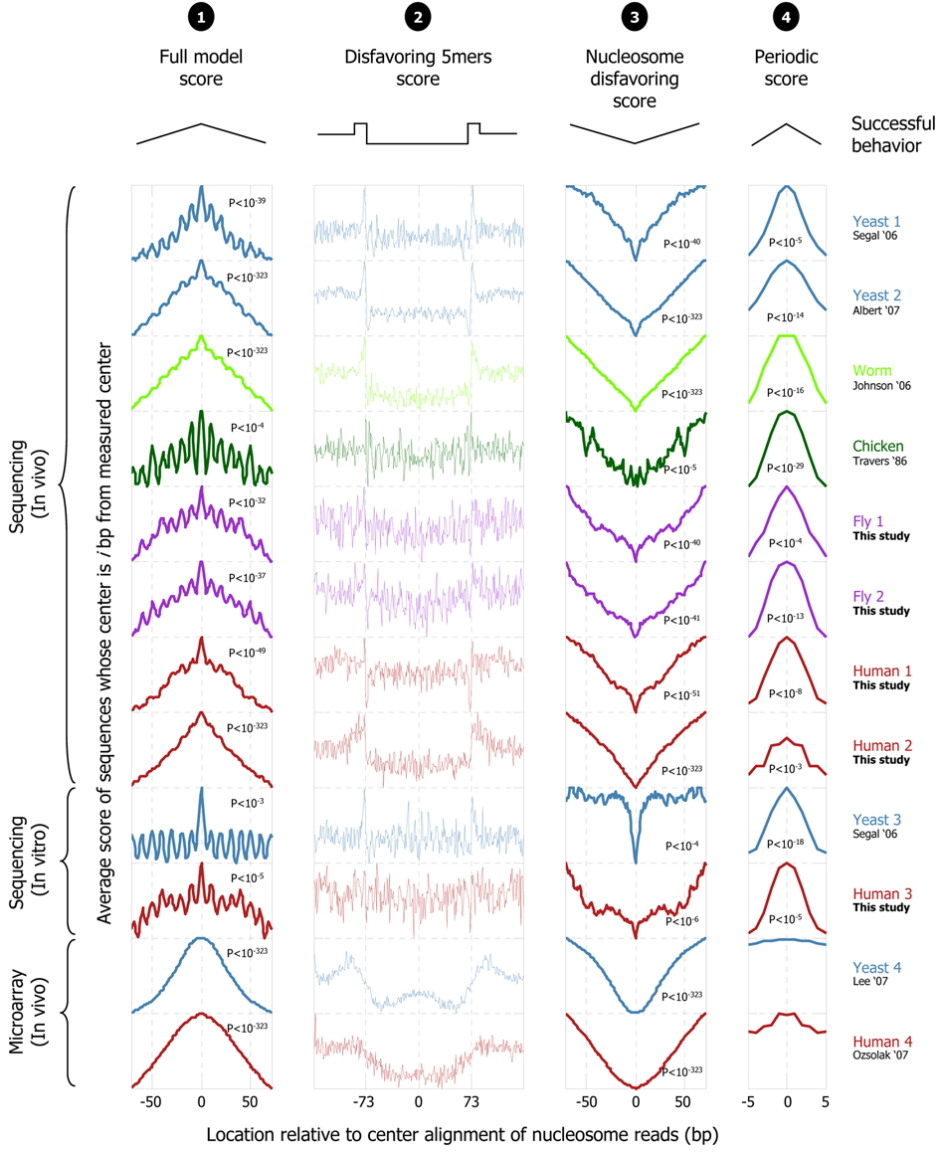
Testing the universality of nucleosome positioning signals across eukaryotes. Our nucleosome model trained from yeast predicts nucleosome locations across several eukaryotes. For various nucleosome collections, including five new ones in fly and human that we isolated here, shown are scores assigned by our full model (“1”; *score*(*S*) from Equation 1 of the Methods section), by only the (position-independent) individual 5-mer component of the nucleosome-disfavoring component (“2”; *P_l_* from Equation 1 above), by the entire nucleosome-disfavoring component of our model (“3”; *P_L_* from Equation 1 above), and by the (position-dependent) periodic component of our model (column “4”; *P_N_* from Equation 1 above). The sequences in each collection were mapped to their respective genome, and the score shown in each column at *x*-axis position *i* is the average score across all sequences in the collection, of the 147 bp (5 bp for column “2”) sequence whose center is *i* basepairs away from the center of the mapped sequence. For the full model (“1”) and nucleosome-disfavoring component (“3”), scores are shown in a window that extends up to 73 bp (half a nucleosome) around the center of the mapped nucleosome. Successful predictions assign their highest (“1”) or lowest (“3”) score at x-axis position zero. The *p*-value represents a student *t*-test that tests whether the distribution of scores in the 40 bp region centered on the mapped nucleosome is significantly higher (“1”) or lower (“3”) than that in the outer 40 bp (20 bp on each end of the mapped nucleosome). For the periodic component (“4”) scores are shown in a 10 bp window around the center of the mapped nucleosome, such that successful predictions assign the highest score at *x*-axis position zero; the *P*-value tests whether the distribution of scores in the 5 bp centered on the mapped nucleosome is significantly higher than that in the outer 6 bp (3 bp on each side, i.e., bp −5,−4,−3 and bp +3,+4,+5 from the center of the mapped nucleosome). Note that in several collections (e.g., worm), the 5-mer component itself (“2”) precisely demarcates the nucleosome positions, by assigning higher scores at the linker regions (more than 73 bp away from the center) compared to the nucleosomal regions (central 147 bp). For all four columns, the *y*-axis is scaled between the minimum and maximum score of the entire 293 bp region centered around the mapped nucleosome.

Notably, in all of the above 12 nucleosome collections, our model assigns, on average, significantly higher scores around the center of the mapped nucleosome locations compared to scores that it assigns to nearby regions, suggesting that the nucleosome positioning signals of yeast are indeed predictive of nucleosome organizations in other eukaryotes (Figure 5). We also separately evaluated each of the two components of our model. We find that in all 10 collections obtained by direct sequencing, the periodic dinucleotide component alone predicts the correct rotational setting to within a 5 bp resolution, since on average, it assigns a higher score to the center of the nucleosome bound sequences in each collection compared to the score that it assigns to positions that are 5 bp away from that center (Figure 5). Similarly, in all 12 collections, the nucleosome disfavoring component of our model alone predicts the correct translational settings of the nucleosomes in each collection, since on average, it assigns a lower score to the center of the nucleosome bound sequences in each collection, compared to scores that it assigns in nearby regions (Figure 5). We also note that the 4th order Markov model alone (this component is the constituent repeating component of the 147 bp nucleosome disfavoring component), readily reveals that its preferred and disfavored 5-mers, learned only from yeast, show similar preferences in these nucleosome collections from higher eukaryotes, such that linkers contain more nucleosome-disfavoring sequences (Figure 5).

The success of our model, which is trained only on yeast nucleosomes, in predicting nucleosome locations across several eukaryotes, suggests that the key nucleosome positioning signals of our model, such as its periodic pattern and 5-mer sequence preferences (and negative preferences), represent nucleosome sequence preferences, and are universal across eukaryotes. Clearly, although this result demonstrates that the nucleosome positioning signals of yeast apply to higher eukaryotes, it does not show that these positioning signals are the only ones that determine nucleosome positioning in higher eukaryotes, and it will be interesting to examine these questions using recent large-scale nucleosome maps in higher eukaryotes [25],[26].

### Poly(dA:dT) Tracts Create Boundary Zones That Demarcate Nucleosome Positions

To better understand the effect of nucleosome-disfavoring sequences on the local depletion of nucleosomes, we focused on the association between nucleosome occupancy and homopolymeric tracts of A or T, termed Poly(dA:dT) elements, since in our data, AAAAA is the 5-mer with the strongest enrichment in linkers (Figure 2B). Several studies examined this relationship [27]–[33], and suggested that Poly(dA:dT) elements may be rigid in vitro [30] and in vivo [28], resulting in a reduced affinity to nucleosomes [34]. These elements are enriched in eukaryotic, but not in prokaryotic, genomes [35], and were shown to have important functions in vivo [27],[29], most likely mediated by their nucleosome disfavoring action [29],[36],[37]. Consistent with this hypothesis, microarray-based maps of yeast [5],[9] and human [24] nucleosomes showed nucleosome depletion over Poly(dA:dT) elements. However, none of these studies focused specifically on quantifying the fold depletion over Poly(dA:dT) elements.

To quantify the fold depletion over a set of Poly(dA:dT) elements of interest, we compare the observed and expected number of nucleosomes that cover these elements. For example, 100 Poly(dA:dT) elements whose combined length is 1,470 bp and that are collectively covered by only one nucleosome read are depleted by 50-fold, since according to the average genome-wide coverage of our map, which is 5 nucleosomes per basepair, we expect these regions to be covered by 50 nucleosome reads. Plotting these fold depletions over Poly(dA:dT) elements of varying lengths, we find large depletions over these elements, that steadily increase with their length (Figure 2E and Figure S4). For example, there is a 12-fold depletion of nucleosomes over the 225 Poly(dA:dT) elements in the yeast genome whose size is at least 17 bp.

We found similarly large fold depletions over Poly(dA:dT) elements with several basepair substitutions and in clusters of short Poly(dA:dT) elements that alternate between strands (Figure 2E and Figure S4). The depletion over these imperfect elements also increases with their length. The large nucleosome fold-depletions over these sequence elements mean that these elements effectively create boundary zones, dividing the genome into discrete chromatin blocks; for simplicity, we henceforth refer to the sequence elements themselves as “boundaries”. The strength of a boundary, defined here as the fold depletion over all of its instances in the genome, can be estimated from DNA sequence alone, based on the length and perfection of its Poly(dA:dT) components. For example, Poly(dA:dT) elements of length 20 with two basepair substitutions have a 6-fold nucleosome depletion (Figure 2E). We find 673 boundary elements in the yeast genome even at fold depletions of more than 10, and these elements are primarily located in non-coding regions (Figure S5).

### Nucleosome Depletion over Boundary Elements Is Unlikely To Be an Artifact

A possible concern is that the nucleosome depletion that we observe over sequence boundaries results from artifacts in our experimental method. Two main concerns arise in this respect. First, the depletion over boundaries may result from biases in the sequencing technology that we employed. Arguing against this, however, are the facts that nucleosome depletion over Poly(dA:dT) elements was observed using the independent technologies of microarrays [5],[9],[10],[24]; using alternative sequencing-based approaches that utilize short reads only and thus do not need to read through a Poly(dA:dT) element itself [13]; and that the effect we see is not restricted to perfect Poly(dA:dT) elements, which could conceivably be problematic [38], but includes elements with many basepair substitutions (Figure 2E) and elements that alternate between Poly-A and Poly-T tracts on each strand (Figure S4). Together, these facts imply that the observed depletions do not result from an inability of our procedure to provide sequence reads from DNA fragments that contain Poly(dA:dT) elements.

A second possible concern may arise from the use of micrococcal nuclease to isolate nucleosomes, since this enzyme was used in both our study and in all of the studies that used microarrays or alternative sequencing-based strategies to map nucleosomes. The concern is that if the sequence specificity of micrococcal nuclease was biased towards Poly(dA:dT) elements, then its use may select against nucleosome DNAs containing these sequence elements. However, such an effect is unlikely because stretches of pure Poly(dA:dT) do not match the known specificity of micrococcal nuclease [24],[39], and hybridizations of micrococcal nuclease-treated naked DNA show little correlation with measured nucleosome locations [9].

**Figure 6:**
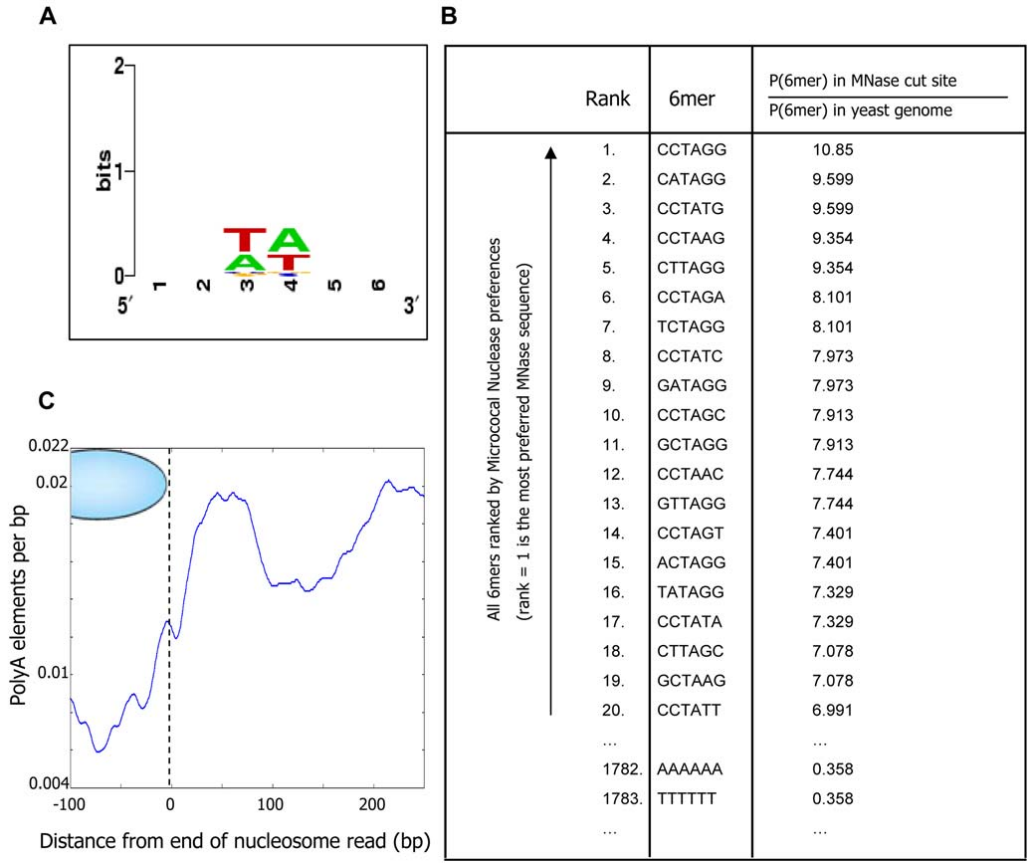
The sequence specificity of micrococcal nuclease is not the cause of nucleosome depletion over Poly(dA:dT) elements. (A) Shown is a standard sequence logo representation of the sequence specificity of micrococcal nuclease, as determined by aligning the ∼1,000,000 cut sites that we obtained in our study. In this standard representation, every position represents the probability distribution over the four possible nucleotides at that position (relative to the yeast genome composition), by the information content contained in that distribution. As can be seen, the information content is low, indicating that although micrococcal nuclease does have detectable sequence specificity, this specificity is low and can thus be found in nearly every small stretch of DNA in the yeast genome. (B) Shown is the ranking of all 4096 possible 6-mers by their preference to be cut by micrococcal nuclease, defined as the ratio between the probability that they appear as a cut site and the probability that they appear in the yeast genome. The top ranking 6-mers are shown, along with the (low ranking) position of AAAAAA and TTTTTT. (C) Shown is the fraction of micrococcal nuclease cut sites in which there is a Poly(dA:dT) element *k* basepairs away from the cut site, when *k* ranges from −100 bp (i.e., 100 bp inside the mapped nucleosome) to 250 bp (outside). For this analysis we took perfect Poly(dA:dT) elements of length 6 or greater. Note that the most likely position for Poly(dA:dT) elements is not at the cut site but rather ∼50 bp from the cut site.

To confirm that nucleosome depletion over Poly(dA:dT) elements is not a result of the sequence specificity of micrococcal nuclease, we examined the ∼1 million cut sites of micrococcal nuclease provided by our data (since we sequenced ∼500,000 individual nucleosomes altogether, and each nucleosome is sequenced in full, thereby providing two cut sites). By aligning all of these cut sites, we find that the sequence specificity in these cut sites is highly similar to that reported previously [39], and that it has very little information content (i.e., the specificity of the nuclease is low, confined mainly to two basepairs). This means that a preferred sequence for micrococcal nuclease can be found in nearly every small stretch of DNA in the yeast genome (Figure 6A). Moreover, ranking all of the 4096 possible 6-mers by their preference to be cut by micrococcal nuclease, defined as the ratio between the probability that they appear as a cut site and the probability that they appear in the yeast genome, we find that AAAAAA is ranked 1782 out of the 4096 possible 6-mers as a micrococcal nuclease cleavage site (Figure 6B), while it ranks number 1 for its observed in vivo nucleosome depletion (Figure 2A). In addition, plotting the distribution of Poly(dA:dT) elements as a function of their distance from all cut sites obtained in our data, we find that the most likely position for Poly(dA:dT) elements relative to cut sites is ∼50 bp from the cut site, which is consistent with the enrichment of Poly(dA:dT) elements in linker DNA regions, but not with the idea that Poly(dA:dT) elements are preferentially cut by micrococcal nuclease (Figure 6C). Thus, the relative lack of nucleosome occupancy over Poly(dA:dT) elements in vivo is not attributable to these sites being preferentially degraded by the micrococcal nuclease.

Taken together, the existing literature, the above analyses, and additional new experimental data that we present in a later section below, strongly suggest that the in vivo depletions that we observe over Poly(dA:dT) elements are not an artifact of our analysis, but a real phenomenon.

### Depletion over Boundaries Likely Results from Their Reduced Nucleosome Affinity

What may cause the observed nucleosome depletion over boundaries? One possible mechanism is through the action of DNA binding proteins that recognize and bind these elements. To date, a single protein in *S. cerevisiae*, called Datin (Dat1p), that recognizes Poly(dA:dT) elements has been identified [40]. The binding specificity of Datin requires at least 9 basepairs of A or T nucleotides, and it appears to be the only DNA binding protein in *S. cerevisiae* that binds Poly(dA:dT) elements, since cell extracts of a Datin deletion yeast strain do not exhibit any detectable protein binding to Poly(dA:dT) elements [40]. However, Datin is unlikely to be the major cause of nucleosome depletion over boundaries, based on the sequence diversity of Poly(dA:dT) elements that we find to be depleted yet that do not match the binding specificities of Datin, on the steady increase of the depletion with the length of the Poly(dA:dT) elements (Figure 2E), and on other studies that concluded that Datin is not important for the function of Poly(dA:dT) elements [28],[29],[36],[37],[41],[42].

**Figure 7:**
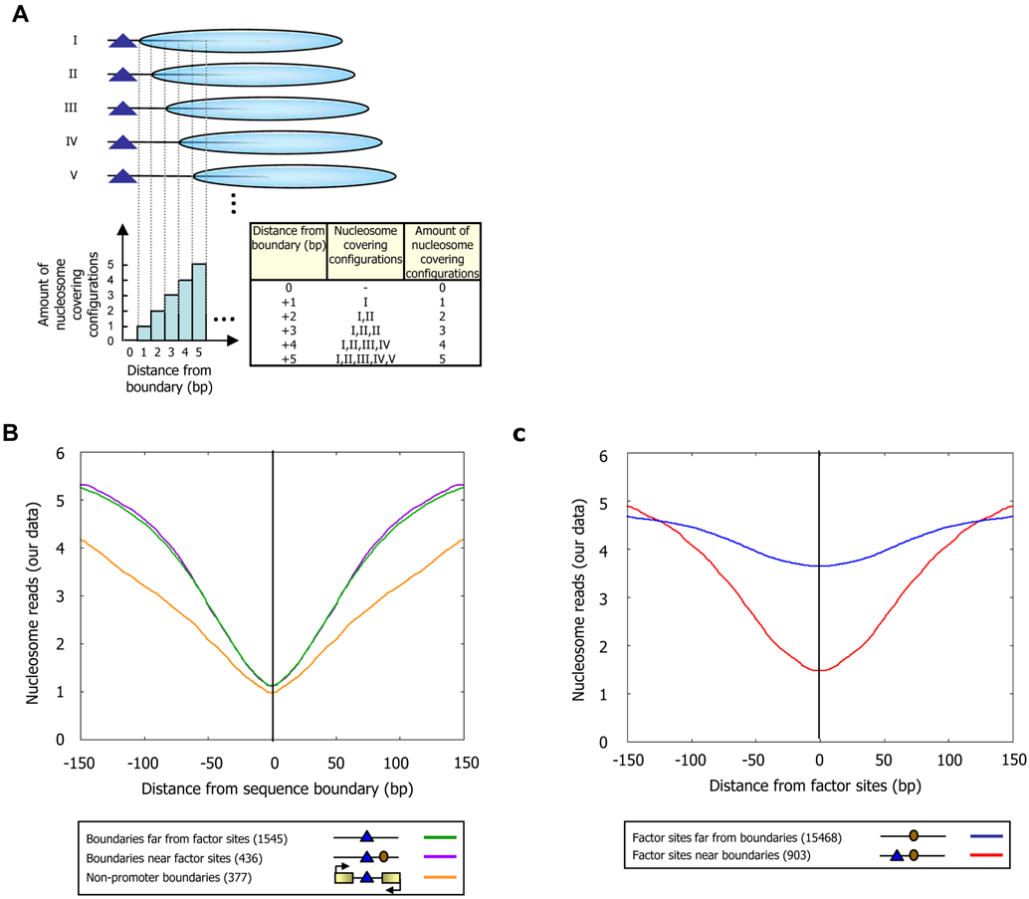
Nucleosome depleted regions are created in the vicinity of Poly(dA:dT) boundaries. (A) A boundary constraint creates, on average, a larger nucleosome-depleted region that extends far into regions flanking the boundary. Shown is a simple example focusing only on the immediate neighborhood of the boundary. All (five) possible nucleosome configurations are illustrated, in which a nucleosome (cyan ovals) can be placed within five basepairs of the boundary (blue triangle). The number and set of nucleosome configurations occupying each of the five basepairs immediately adjacent to the boundary are shown in the graph and table, respectively. If all configurations are equally likely, then basepairs closer to the boundary will exhibit lower nucleosome occupancy. (B) Boundaries exhibit strong and long-range nucleosome depletion regardless of whether they are near transcription factor binding sites or whether they are in promoters or non-promoter intergenic regions. Shown is the average number of nucleosome reads in our data at locations *k* (for *k* = 1,2,…,150) basepairs away from boundaries (strength >5) that are: more than 30 bp from any factor site (green); within 30 bp of a factor site bound by its cognate factor [47] (purple); in intergenic regions that are not promoters (orange). The strength of a boundary is defined by properties of the DNA sequence of the boundary, based on the length and perfection of the Poly(dA:dT) components of the boundary (see Methods). Plots are symmetric by construction. (C) Boundaries enhance the accessibility of transcription factors to cognate sites. Shown is the average number of nucleosome reads in our data at locations *k* (for *k* = 1,2,…,150) basepairs away from annotated factor binding sites bound by their cognate factor [47] that are: more than 30 bp from any boundary (boundary strength >5) (blue); within 30 bp of any boundary (strength >5) (red). Plots are symmetric by construction.

Another possibility is that the binding of transcription factors to sites near the boundaries causes nucleosome depletion over boundaries. Indeed, such an effect is to be expected on thermodynamic grounds; the question is the relative significance of this effect. To test this, we compared the nucleosome occupancy over boundaries that are near factor binding sites, to that over boundaries that are far from factor sites. We find strong nucleosome depletion over boundaries regardless of whether or not they are near factor sites (Figure 7B). This result is not sensitive to binding site annotations, since we find a similar strong depletion over boundaries in intergenic regions that are not promoters, thought to be largely devoid of factor sites (Figure 7B). These results suggest that transcription factor binding is not the main cause of nucleosome depletion over the boundary sequences.

A remaining alternative is that Poly(dA:dT) elements themselves intrinsically disfavor nucleosome formation. This possibility was suggested previously, on the basis of studies done on a handful of genes [29]–[31],[36],[37], though other single gene studies [43],[44] concluded that nucleosome exclusion by Poly(dA:dT) elements cannot account for the full effect of Poly(dA:dT) elements. Studies of the structure and mechanics of Poly(dA:dT) elements further support that these elements act through nucleosome exclusion, since these tracts may be mechanically stiff and thus resist wrapping into nucleosomes [30].

**Figure 8:**
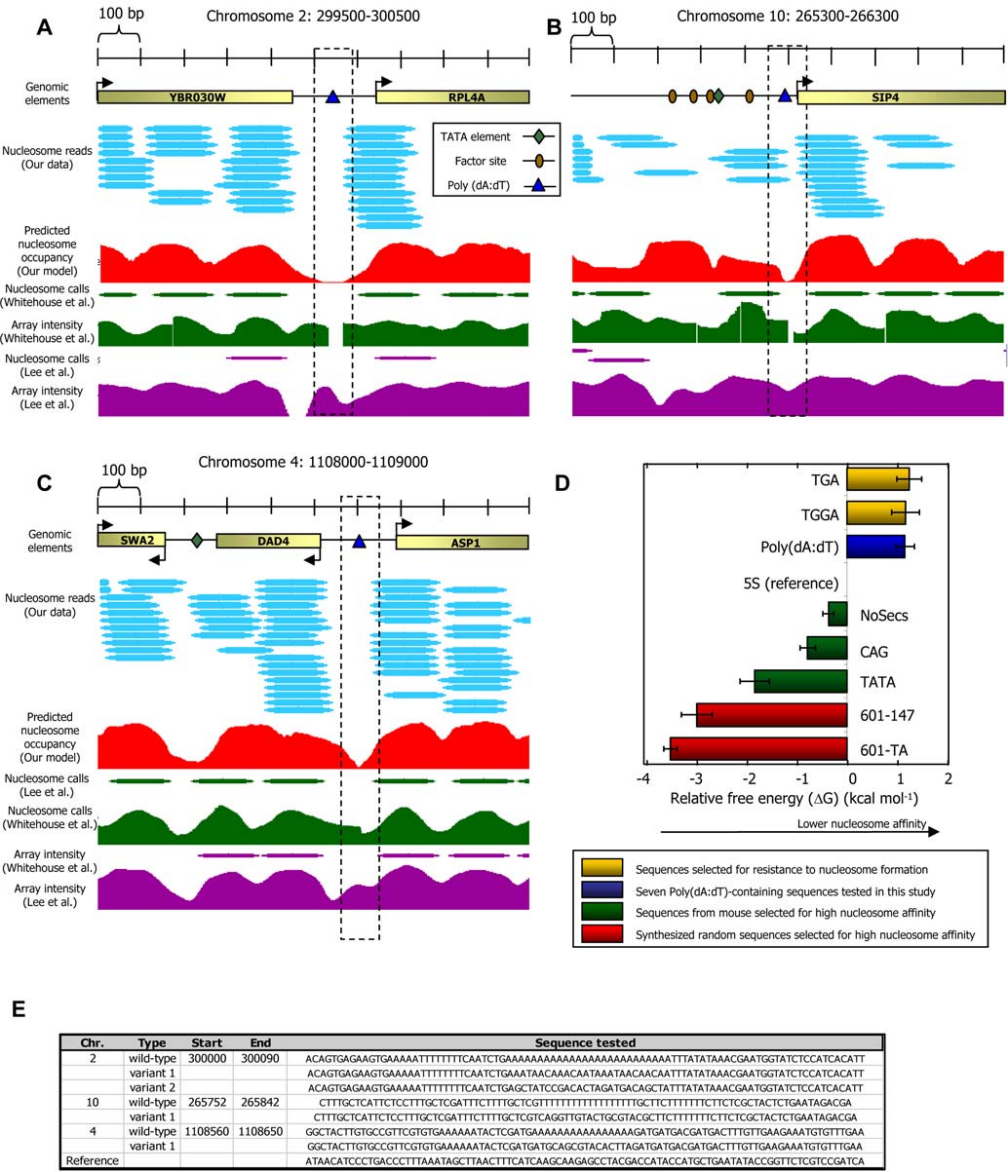
Poly(dA:dT) elements have a reduced affinity for nucleosome formation in vitro. (A–C) Experimental maps of nucleosome occupancy at three genomic loci for which we measured the relative nucleosome affinity of Poly(dA:dT)-containing sequences (blue triangles). Every cyan oval represents the genomic location of one nucleosome that we sequenced in its entirety. Also shown is the average nucleosome occupancy per basepair predicted by the sequence-based nucleosome model that we developed here (red), the raw hybridization signals of two microarray-based nucleosome maps [5],[10] (green and purple traces), and the locations of nucleosomes that were computationally inferred from these hybridization signals [5],[10] (green and purple ovals). Annotated genes [63], transcription factor binding sites [47], and TATA sequences [53] in the region are indicated. (D) Poly(dA:dT)-containing sequences have low nucleosome affinities. Shown are measurements of relative affinity for nucleosome formation of seven Poly(dA:dT)-containing sequences (blue bar; shown are mean and standard deviation for seven measured sequences: three boundary regions from yeast that each contain multiple Poly(dA:dT) elements, and four sequence variants that disrupt one of the Poly(dA:dT) elements in each sequence). For comparison, also shown are the relative affinities of sequences selected for their relative resistance to nucleosome formation [45] (yellow bars), and of sequences selected for their high nucleosome affinity from the mouse genome [18] (green bars) and from chemically synthesized random sequences [7],[19] (red bars). All results are presented relative to the 5S reference sequence, defined as 0. (E) The sequences of the Poly(dA:dT)-containing elements of (a–c) that we measured, along with their chromosomal locations.

If nucleosome exclusion is the primary mechanism by which Poly(dA:dT) elements exert their effect, then we might also expect these elements to show a reduced affinity for nucleosome formation in vitro. One study addressed this question, and demonstrated that incorporating a perfect Poly-A(16) element into a (non-natural) DNA sequence disfavors nucleosome formation, with an effect of about two-fold on DNA accessibility [31]. To examine whether natural boundary sequences also exhibit reduced nucleosome affinity in vitro, we selected three Poly(dA:dT)-containing regions from the yeast genome that each contain multiple Poly(dA:dT) elements and measured the relative affinities of these regions for nucleosome formation along with the relative affinities of four sequence variants that disrupt one of the Poly(dA:dT) elements in each sequence. Like many of the other Poly(dA:dT) elements in the genome, the Poly(dA:dT) elements that we selected exhibit nucleosome depletion in vivo (Figure 8A–C). Consistent with earlier measurements [31], we find that all seven Poly(dA:dT)-containing sequences have significantly reduced affinities, comparable to affinities of DNA sequences that were selected for their ability to resist nucleosome formation [45] (Figure 8D and 8E). These relative affinity measurements for nucleosome formation were performed as previously described [2],[7].

We also examined a systematic study of in vitro nucleosome reconstitution on ∼10 kbp from the β-Lactoglobulin locus of sheep [8], and found strong nucleosome depletion over the one Poly-T(13) element in the locus (Figure S6).

Taken together, these results demonstrate that sequence boundaries have an intrinsically reduced affinity for nucleosome formation. Thus, our new in vitro measurements of nucleosome formation on boundaries, combined with the conclusions reached by previous studies, and with the conclusion that Poly(dA:dT)-binding proteins and transcription factors cannot account for the in vivo depletion over Poly(dA:dT) elements, strongly suggest that the large in vivo depletion over Poly(dA:dT) elements is the consequence of a nucleosome-disfavoring character of these elements.

### Boundaries Enhance the Accessibility of Transcription Factors to Their Sites

What may be the function of sequence boundaries? In the extreme case, a strong boundary that cannot be occupied by a nucleosome creates, on average, a nucleosome-depleted region centered on but larger than the boundary itself, simply because there are a smaller number of different nucleosome configurations in which basepairs that are close to the boundary can be occupied by a nucleosome, compared to basepairs located further away from the boundary. For example, a basepair immediately flanking the boundary can only be occupied by the one configuration in which a nucleosome is placed immediately adjacent to the boundary, whereas a basepair located 5 bp from the boundary can be occupied by any of 5 different nucleosome configurations (Figure 7A). Ignoring the nucleosome sequence preferences for a moment, and assuming for simplicity that all allowed nucleosome positions are equally likely, then, in the above example, the basepair immediately flanking the boundary is 5-times less likely to be occupied by a nucleosome, compared to the basepair located 5 bp away from the boundary. Thus, the mere presence of a boundary acts as a force that, on average, creates a nucleosome-depleted region extending into the adjacent DNA [46].

**Figure 9:**
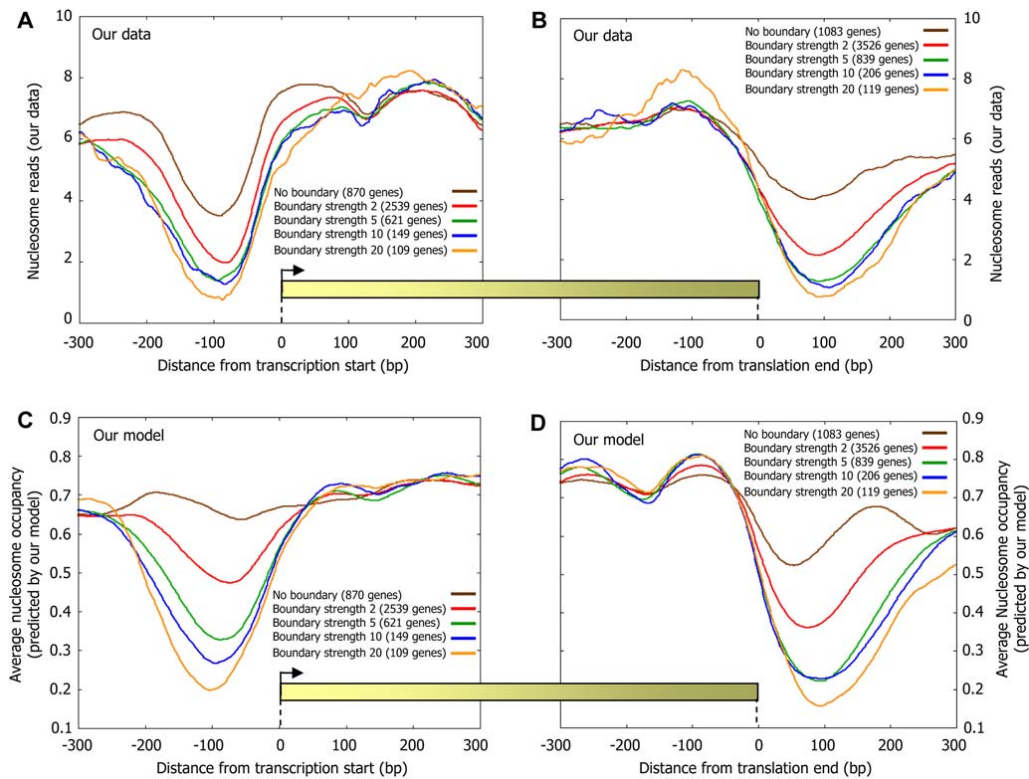
The level and length of nucleosome depletion around gene start and gene end sites correlate with boundary strength. (A) Boundaries were classified into five groups by their nucleosome fold depletion (strength) using sequence rules (see Methods), and every gene was annotated by the classification of the strongest boundary that it has in the 200 bp region upstream of its transcription start site. Shown is the average number of nucleosomes per basepair around the transcription start site of genes from each of the four boundary classification groups. (B) Same as (A), but when annotating each gene by the classification of the strongest boundary that it has in the 200 bp region downstream of its translation end site (translation end site was chosen since transcription end sites are poorly annotated). Note that for a given boundary class, the corresponding genes in (A) are distinct from the corresponding genes in (B). (C,D) Same as (A) and (B), but plotting the average nucleosome occupancy predicted by the sequence-based nucleosome positioning model that we developed here. Predictions are generated in a cross validation scheme, such that the predicted nucleosome occupancy across each chromosome is computed by a model that was learned using only the nucleosome data of all the other chromosomes.

Based on the above reasoning, we hypothesized that the flanking regions of our above Poly(dA:dT) boundaries will be depleted of nucleosomes, and we expect the strength of the effect to increase with the strength of the boundary. Indeed, examining the nucleosome occupancy in the vicinity of boundaries, we find large levels of nucleosome depletion even 50 bp away from a boundary, regardless of whether or not the boundary is located close to a transcription factor binding site, and whether or not the boundary is located in a promoter region or in intergenic regions that are not promoters (Figure 7B). Moreover, examining the distribution of boundaries around transcription start sites where previous studies [5],[9],[13] found a stereotyped nucleosome depleted region, and around translation end sites where similar depletions were observed [11],[12],[26], we find that both the depletion level and length of these depleted regions strongly correlate with the boundary strength (Figure 9A and 9B). As expected, these differing nucleosome organizations around both transcription start sites and translation end sites are accurately predicted by our sequence-based model for nucleosome positioning (Figure 9C and 9D).

These results are consistent with the theoretical analysis of Kornberg and Stryer [46], although, their boundary constraint was thought to be due to transcription factors, whereas we show that a boundary constraint arises also simply from the presence of Poly(dA:dT)-based sequence elements, through their reduced affinity for nucleosome formation. Our results thus suggest that relatively large open chromatin regions can be accurately predicted simply by the presence of Poly(dA:dT) elements, consistent with the suggestion that boundaries such as Poly(dA:dT) elements account for many aspects of the in vivo nucleosome organization [9],[12].

If boundaries indeed cause nucleosome depletion at their flanking regions, then boundaries may enhance the accessibility of transcription factors to binding sites that are located close to the boundary. Indeed, we find strong nucleosome depletion over factor sites that are near boundaries, compared to a much weaker depletion over factor sites that are far from boundaries (Figure 7C), suggesting that nucleosome depletion over many factor sites is partly encoded through the sequence preferences of nucleosomes, by the nucleosome-disfavoring action of Poly(dA:dT) elements. These results are consistent with studies done at a few loci, which suggested that Poly(dA:dT) elements may generally function to enhance the accessibility of transcription factors to their cognate sites [27],[29].

**Figure 10:**
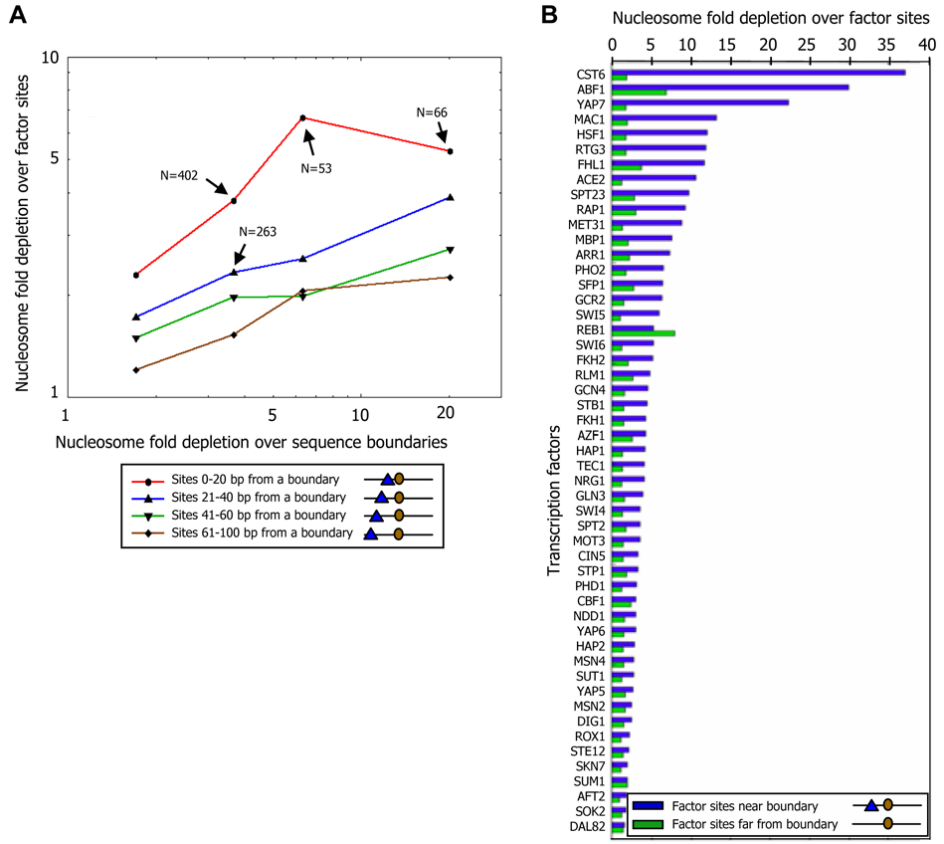
Boundaries enhance the accessibility of transcription factors to their cognate binding sites. (A) Nucleosome depletion over factor sites increases with their proximity to, and with the strength of, boundaries. Shown is the combined nucleosome fold depletion over factor sites (*y*-axis) that are within a certain range of distances from boundaries that themselves have a particular nucleosome fold depletion (boundary strength; *x*-axis). Plots are shown for four different ranges of factor-boundary distances and for the four boundary strength groups of nucleosome fold depletions that we defined based on sequence rules (see Methods). (B) Factor binding sites near boundaries are depleted of nucleosomes. For each factor, shown is the combined nucleosome fold depletion over its annotated sites [47],[67] that are within 30 bp from a boundary whose fold depletion is at least 5 (blue bars), and over the rest of its sites (green bars). The combined fold depletion of a set of genomic elements is the ratio between their expected and observed nucleosome coverage (see Methods).

We next asked whether nucleosome depletion over factor sites depends on the boundary strength and factor-boundary distances. Notably, the level of nucleosome depletion over factor sites increases significantly with both the strength of the boundary and its proximity to factor sites (Figure 10A). Specifically, for 50 of 51 factors for which more than 10 sites are annotated [47], we find stronger nucleosome depletion at the subset of its sites that are near boundaries compared to its other sites (Figure 10B). The only exception is Reb1, a highly abundant factor that possesses ATP-independent chromatin remodeling activity [48]. Taken together, our results demonstrate that boundaries enhance the accessibility of transcription factors to their cognate sites, by depleting nucleosomes from the adjacent DNA, with the magnitude of such depletion increasing with both the strength of the boundary and its proximity to the factor site.

### Two Different Types of Regulation by Chromatin in Yeast Promoters

**Figure 11:**
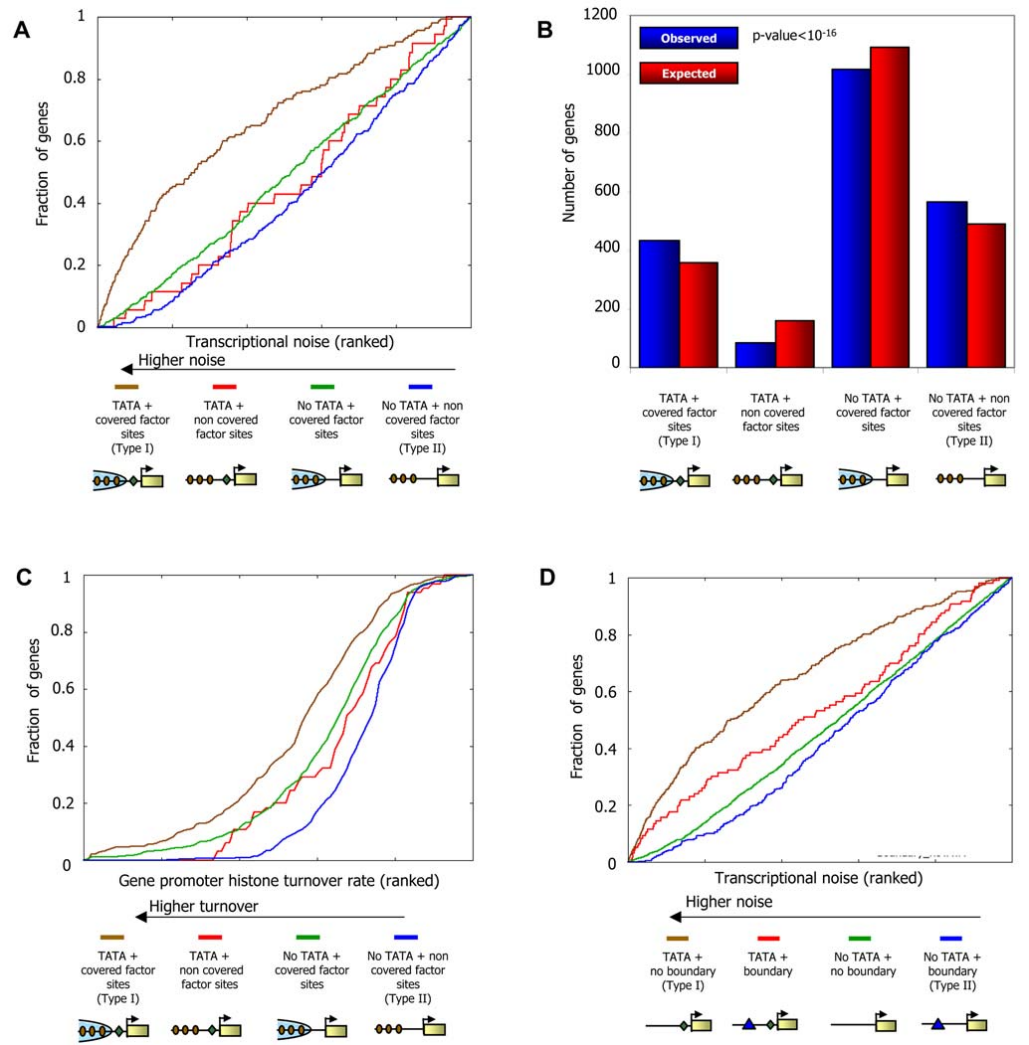
Two different types of regulation by chromatin in yeast promoters. (A) Promoters with TATA elements and whose binding sites are located in regions covered by nucleosomes exhibit large transcriptional noise. Genes were divided into four groups based on the presence or absence of TATA elements [53], and by whether their binding sites are covered by nucleosomes or are nucleosome-depleted as measured in our map (see Methods). For each group of genes, shown is the fraction of its genes (*y*-axis) whose noise level is within the *k* most noisy genes (*x*-axis; expressed as fraction), for all possible values of *k*. Measurements of transcriptional noise are available for 2197 genes [49] and are presented in their ranked value. (B) Yeast promoters are enriched with architectures that are associated with high- and low-noise. For each of the four gene sets from (A), shown is the actual number of genes in each set (red bar) compared to the expected number of genes in each set (blue bar). The number of genes in the two extreme promoter types (type I: leftmost columns, genes with TATA elements and nucleosome-covered factor sites; type II: rightmost columns, genes without TATA elements and with nucleosome-depleted factor sites) is significantly more than would be expected just from the counts of the number of genes with/without TATA elements and with nucleosome-depleted/nucleosome-covered sites (*P*<10^−16^, hypergeometric test). (C) Promoters with TATA elements and whose binding sites are located in regions covered by nucleosomes as measured in our map exhibit large degrees of histone turnover. For each of the four gene sets from (A), shown is the fraction of its genes (*y*-axis) whose histone turnover level [55] is within the *k* promoters with the largest degree of histone turnover (*x*-axis; expressed as fraction), for all possible values of *k*. Measurements of histone turnover are presented in their ranked value. (D) Promoters with distinct transcriptional noise characteristics can be predicted from sequence alone. Same as (A), but when dividing genes using only sequence information, based on the presence of Poly(dA:dT)-boundaries and TATA elements. Genes were divided into four groups based on the presence of TATA elements [53], and by whether or not they have a boundary of strength >5 within the 200 bp region upstream of their transcription start site (where the boundary strength is defined based on DNA sequence alone).

We hypothesized that since factor binding sites near boundaries are depleted of nucleosomes, factors could bind such sites in promoters with little or no competition with nucleosomes, leading to a homogeneous cell population with relatively low cell-to-cell expression variability, or transcriptional noise. In contrast, since steric hindrance may not permit simultaneous binding by factors and nucleosomes, factors that bind sites that are far from boundaries may need to compete with nucleosomes for access to the DNA. Such a competition may result in a mixed population comprising both cells in which a nucleosome covers the factor's site and the promoter is inactive, and cells in which that nucleosome is displaced and the promoter is active. To test this hypothesis, we utilized a dataset [49], which for the majority of the genes in yeast, used a GFP-tagged strain to measure their protein expression variability in single-cells. Since they are easier to obtain, such measurements of variability at the protein level are typically used as a proxy for variability measurements at the RNA level [49]–[51]. This approach is justified by the experimental observation that variability in protein expression is dominated by variability in RNA levels [49]. Using these data, we compared the noise of promoters in which the sites [47] are covered by nucleosomes, to the noise of promoters in which the sites are not covered. Indeed, the former promoter set exhibits significantly more noise (*P*<10^−5^, Kolmogorov-Smirnov test). A similar model, in which high noise promoters are those where nucleosomes compete successfully with transcription factors, was suggested and validated for the Pho5 gene [51]. That model further suggested that the presence of TATA sequences should confer even more noise, presumably through facilitation of transcription re-initiation [51],[52]. Thus, under this noise model, we expect, and indeed find, that within each of our two promoter sets above, the presence of TATA [53] elements further increases transcriptional noise (Figure 11A).

**Figure 12:**
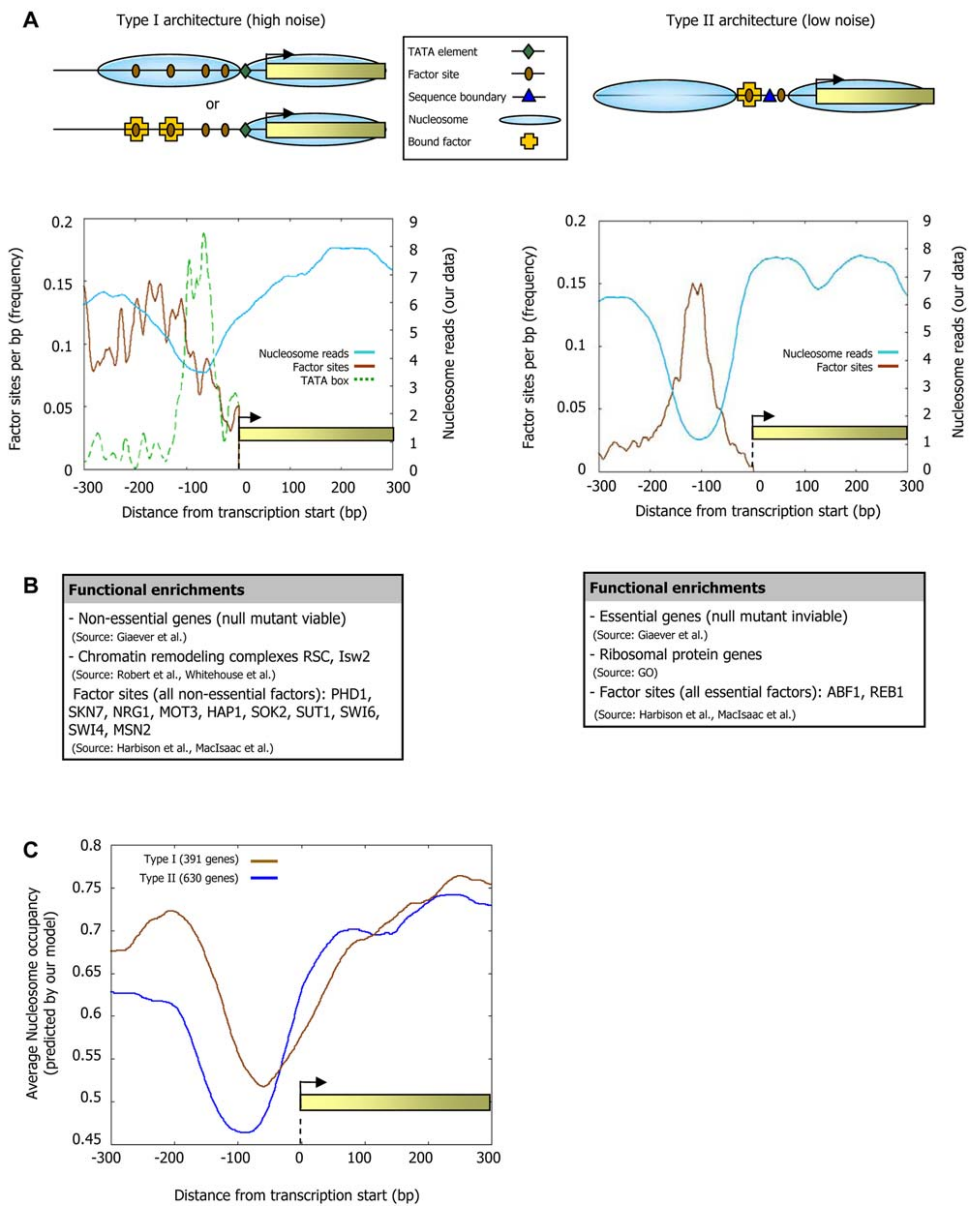
Type I and Type II promoters have distinct architectures. (A) Shown is a schematic illustration of promoter architectures for the two extreme types of promoters from Figure 11A. The schematic illustrates that in the high noise (Type I, left column) promoters, factor binding sites are measurably occupied by both their cognate factors and nucleosomes (in a cell population), suggesting that their high noise results from competition between nucleosomes and factors for DNA access. In contrast, the low noise (Type II, right column) promoters exhibit a characteristic nucleosome-depleted region upstream of the transcription start site in which bound factor sites are highly concentrated. Also shown is the average number of nucleosome reads in our data (cyan), and the distribution of factor sites (brown) and TATA elements (green, only for Type I promoters), around the transcription start site of the genes in each of the two extreme types of promoters from (A) (left column, Type I promoters; right column, Type II promoters). (B) Genes of the high- and low-noise promoter classes exhibit distinct functional enrichments. Shown is a selected list of functional categories that are significantly enriched (*P*<10^−5^) in the set of genes associated with each promoter type (see Figure S7 for the full list and details of all enrichments). (C) The distinct nucleosome organizations in high- and low-noise promoters can be predicted from DNA sequence. Shown is the average nucleosome occupancy predicted by the sequence-based model for nucleosome positioning that we developed here, for each of the two promoter types in (A).

We further examined those promoters having TATA elements and nucleosome-covered factor binding sites, and those promoters lacking TATA elements and having nucleosome-depleted factor binding sites, since these promoter sets are the most and least noisy promoters, respectively (Figure 11A), and they each have more genes than would be expected (Figure 11B). Intriguingly, in addition to their differential noise, we also find distinct promoter architectures and nucleosome dynamics in these two promoter types. Type I promoters, which contain TATA elements and whose sites are nucleosome-covered, have many factor sites spread across the promoter region, a weaker signal of nucleosome depletion at the typical nucleosome depleted region (NDR), and are enriched in targets of condition-specific factors and non-essential genes (Figure 12A and 12B and Figure S7). These promoters are targets of chromatin remodeling complexes [54] and their rate of histone turnover [55] is significantly high (Figure 11C), consistent with an ongoing dynamic competition between nucleosome assembly and factor binding. In contrast, type II promoters, which are TATA-less and whose sites are nucleosome-depleted, have strong nucleosome depletion, many boundary elements at the typical NDR, low histone turnover, and an overall smaller number of factor sites but with a high preference for these sites to be located at the NDR (Figure 12A). Type II promoters are enriched in essential genes and in ribosomal protein genes, the latter presumably owing to the fact that these proteins are highly expressed and are required stoichiometrically in a large complex, thereby conferring a benefit to regulation with low noise (Figure 12B).

While our paper was in review, analysis of nucleosome occupancy data resulted in a similar two-class partition of yeast promoters [56]. We find that our sequence-based nucleosome–DNA interaction model accurately predicts the different nucleosome organizations observed for each promoter type, suggesting that their distinct nucleosome architectures are partly encoded in the genome through the sequence preferences of nucleosomes (Figure 12C). In fact, we can distinguish low- and high-noise promoters using only sequence information, by partitioning promoters according to the presence of our Poly(dA:dT)-boundaries and TATA elements (Figure 11D). Taken together, our results point to a strong association between chromatin and transcriptional noise at the genome-wide level, as suggested on the basis of one gene [51], and further uncover two distinct types of chromatin architectures by which high or low noise may be implemented in yeast promoters.

### Nucleosome Positioning Signals May Play a Role in Efficiency of DNA Replication

**Figure 13:**
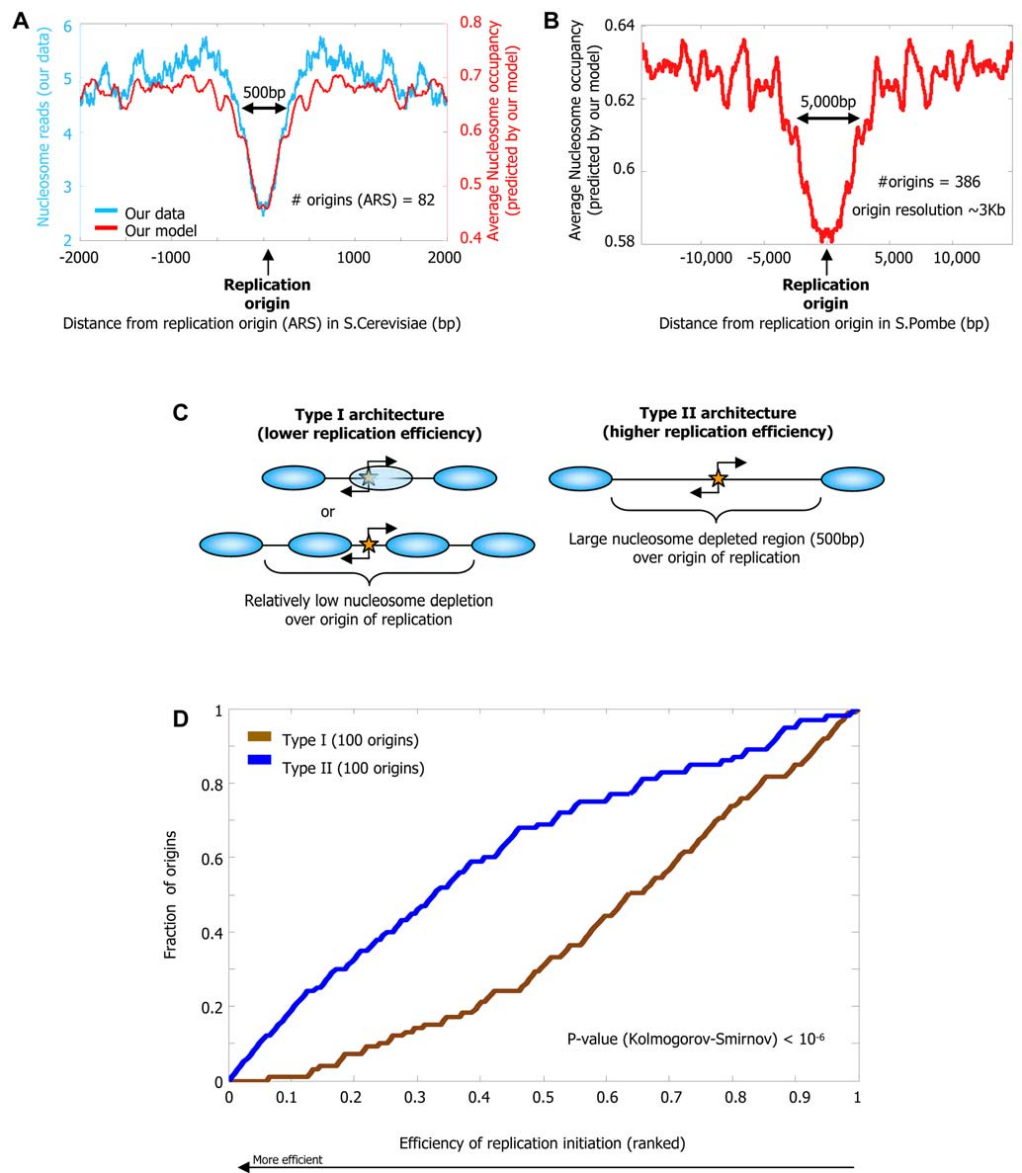
Nucleosome positioning signals may explain DNA replication efficiency. (A) Nucleosomes are depleted from origins of DNA replication in *S. cerevisiae*. Shown is the average number of nucleosome reads in our data (cyan) per basepair around 82 annotated origins of replication from yeast [63]. Note that the typical length of the nucleosome depleted regions is greater around replication origins than it is around transcription start sites (e.g., compare to the length of the depleted region from Figure 9A and 9B). Also shown is the average nucleosome occupancy predicted by the nucleosome positioning model that we developed here (red), per basepair around the same 82 origins. (B) Nucleosome depletion is predicted around replication origins from *S. pombe*. Shown is the average nucleosome occupancy predicted by our nucleosome positioning model (red), per basepair in the vicinity of 386 annotated origins of replication from *S. pombe* [61]. The exceptionally large length of the nucleosome depleted regions around these replication origins may reflect the lower resolution with which *S. pombe* origins are mapped (∼3 Kb), compared to their *S. cerevisiae* analogs. (C) Shown is a schematic illustration of replication origins with low and high replication efficiency. The schematic illustrates that in the low efficiency origins (“type I”, left column), binding sites for the replication machinery are measurably occupied by both their replication factors and nucleosomes (in a cell population), suggesting that their low efficiency results from competition between nucleosomes and factors for DNA access. In contrast, the high efficiency origins (“type II”, right column) exhibit a characteristic nucleosome-depleted region that allows the replication machinery to access the origins and replicate the DNA with high efficiency. (D) Replication origins from *S. pombe* that have large nucleosome depleted regions are utilized with greater efficiency. We computed the average (predicted) nucleosome occupancy in 500 bp windows within the 3 kb region surrounding each of the 386 annotated origins from (B). With each replication origin, we associated the lowest nucleosome occupancy in any of its 500 bp windows. The 3 kb region was selected since the data on replication efficiency have a ∼3 kb resolution [61]; 500 bp windows were selected since these are the typical lengths of the nucleosome depleted regions over origins in *S. cerevisiae*, where origins are mapped with greater accuracy. Using these computed lowest nucleosome occupancies for origins, we grouped together the 100 origins that have the highest of these values (type I), and the 100 origins that have the lowest of these values (type II). For each of these two groups, shown is the fraction of its origins (*y*-axis) whose efficiency of replication initiation as measured in [61] is within the *k* most efficient origins (*x*-axis; expressed as fraction), for all possible values of *k*. Measurements of efficiency of replication initiation are presented in their ranked value.

**Figure 14:**
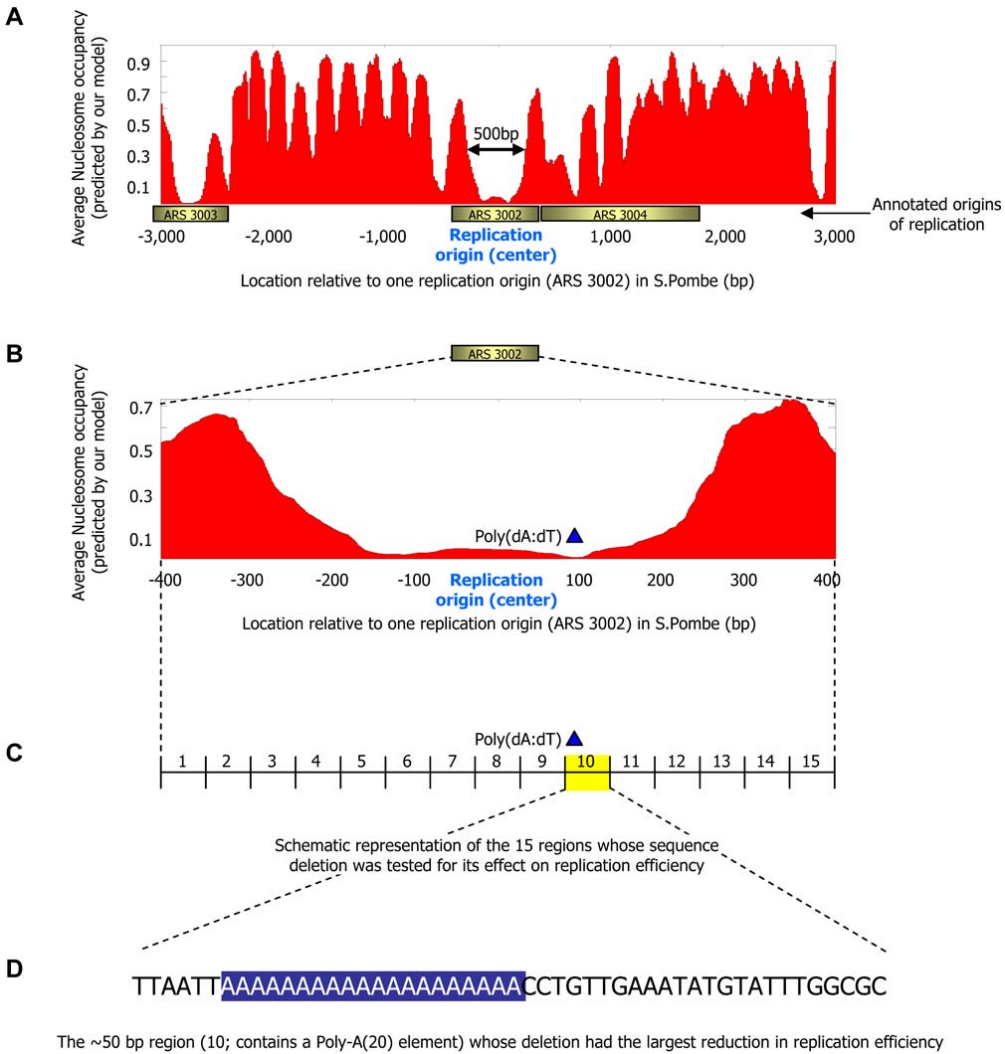
Deletion of a Poly(dA:dT) element from a replication origin results in a reduction in replication efficiency. (A) Shown is the average (predicted) nucleosome occupancy of the nucleosome positioning model that we developed here (red) at the 6 kb region surrounding the one replication origin from *S. pombe* (“ARS 3002”) that was studied in the systematic sequence deletion study of [60]. Our model predicts a nucleosome depleted region around the replication origin (“ARS 3002”). Annotated replication origins in the region were taken from [60] (B) Same as (A), but only around the 815 bp region of the studied origin (“ARS 3002”). (C) Schematic representation of the 15 regions of length ∼50 bp that were each deleted in the study of [60]. The replication efficiency of each of these 15 regions was tested in [60], and it was found that of all 15 regions, deletion of region 10 (which contains a Poly(dA:dT) element) resulted in the largest reduction in replication efficiency. (D) The DNA sequence of region 10 from [60]. The Poly(dA:dT) element is indicated.

Finally, analogous to the cell-to-cell variability observed in gene expression [49], DNA replication origins also exhibit variability, with some origins initiating replication in most cell divisions and others initiating only occasionally. We examined whether this variability can be partly explained by differing nucleosome positioning signals in the two types of origins. In general, DNA replication origins are A/T- and Poly(dA:dT)-rich [57],[58] and thus may disfavor nucleosome formation. Indeed, we find an overall (both measured by our data and predicted by our model) nucleosome depletion around replication origins in *S. cerevisiae* (Figure 13A), and similar (predicted) depletion around origins in *S. pombe* (Figure 13B). Consistent with the hypothesis that competition with nucleosomes may affect the efficacy of replication initiation [59], a systematic sequence deletion study [60] around one replication origin in *S. pombe* found that deletion of a strong nucleosome-disfavoring element (Poly-A(20)) resulted in the largest reduction in replication efficiency (Figure 14). Similarly, for *S. pombe*, where data on efficiency of replication initiation are available [61] (such data are not available for *S. cerevisiae*), we find on a genome-wide scale, that replication origins with lower (predicted) nucleosome occupancy initiate replication with higher efficiency (*P*<10^−6^; Figure 13C and 13D).

## Discussion

Recently, progress was made in understanding the way in which nucleosome organizations are encoded in the DNA sequence. Separately, many studies revealed that the detailed positions of nucleosomes have critical roles in transcription factor binding and transcriptional regulation. Here, we present advances on both questions, and identify a link between the two, by showing that distinct transcriptional behaviors are partly encoded through the genome's intrinsic nucleosome organization.

Utilizing the high spatial accuracy of the full length sequence-based map of yeast nucleosomes, we improve our understanding of the intrinsic genomic signals that determine nucleosome occupancy, and find that these signals include important contributions both from periodicities of specific sequences along the nucleosome and from sequences that are generally disfavored by nucleosomes regardless of their position along the nucleosome. When combining these signals into a probabilistic sequence-based nucleosome–DNA interaction model, we achieve high accuracy in predicting nucleosome organizations in vivo, even across new nucleosome collections that we isolated from fly and human, suggesting that nucleosome positioning signals are universal. Among the nucleosome disfavoring signals, variants of Poly(dA:dT) sequences are most dominant. We find thousands of such Poly(dA:dT) elements in the yeast genome with large levels of nucleosome depletion, where the depletion level can be estimated from DNA sequence alone, suggesting that these elements act as boundaries to exclude nucleosome formation.

Our results suggest that the yeast genome utilizes these nucleosome positioning signals to encode both relatively open (nucleosome-depleted) chromatin architectures that result in low transcriptional noise, and relatively closed (nucleosome-covered) chromatin architectures that result in high noise. We show that closed chromatin architectures may be important for encoding condition-specific transcriptional programs. We find that the effect of chromatin on the activity of a binding site is determined mainly by whether the site is located in an encoded open or closed chromatin region. We hypothesize that such a mechanism may allow the same factor to regulate different targets with different activation kinetics, by having some of its sites located in encoded open chromatin regions and others of its sites at regions encoded to be in closed chromatin architectures. Similarly, we find that DNA-encoded open and closed chromatin architectures may impact the efficiency of DNA replication initiation. It will be interesting to identify other chromosome functions where nucleosome positioning signals play a role and to see whether similar rules apply in higher eukaryotes.

### URLs

For our data, model and genome-wide occupancy predictions in yeast, worm, fly, mouse, and human, and sequences provided by researchers, see http://genie.weizmann.ac.il/pubs/field08. Our results are also viewable in Genomica (http://Genomica.weizmann.ac.il).

## Methods

### Parallel Sequencing of Yeast Nucleosomes and Data Processing

Mono-nucleosomes were extracted from log-phase yeast (*Saccharomyces cerevisiae*) cells using standard methods. The DNA (pooled together from eight independent biological replicates) was extracted, and protected fragments of length ∼147 bp were sequenced using 454 pyrosequencing. Each of the resulting 503,264 sequence reads was mapped to the yeast genome using BLAST [62] requiring at least 95% identity. Sequences were further filtered by requiring that they: map to a unique genomic location; are of length 127–177 bp; do not overlap the ribosomal RNA locus (chromosome 12: 451550–490540 bp). The resulting 378,686 nucleosomes constitute the nucleosome collection used in all of our analyses. We used a sequencing technology whose reads are ∼200 bp in length, and thus, each of the nucleosomal DNA fragments was read in full. These full sequence reads allow us to map both ends of each nucleosomal DNA fragment to the genome, without having to infer its other end, as is the case when using sequencing technologies with shorter reads that map only one nucleosome end.

### Sequencing of Human and Fly Nucleosomes and Data Processing

Two fly and one human in vivo nucleosome collections were obtained from fly (*Drosophila Melanogaster*, S2 cells) and human (HeLa cells). Nuclei were prepared using standard methods, and the chromatin digested to primarily mononucleosomes using micrococcal nuclease. The DNA was extracted, and protected fragments of length ∼147 bp were cloned and sequenced as described [2]. An additional human in vivo nucleosome collection that is strongly enriched in G/C nucleotides (60% G/C) was obtained by digesting the isolated human mononucleosomal DNA with two restriction enzymes: Mse I, and Tsp509, with specificities of TTAA and AATT, respectively. DNA fragments remaining ∼147 bp in length following these digestions were gel purified, cloned, and sequenced. A collection of human in vitro nucleosome sequences was obtained as described previously from yeast [2] except using human genomic DNA instead of yeast DNA [7]. The resulting sequences from each experiment were mapped to their respective genomes using BLAST [62] requiring at least 97% identity. Sequences were further filtered by requiring that they map to a unique genomic location and have a length in the range 142–152 bp. The resulting sequences constitute the three fly and three human nucleosome collections used in our analyses and they have 99 (fly 1; in vivo), 170 (fly 2; in vivo), 329 (human 1; in vivo), 208 (human 2; in vivo G/C), and 176 (human 3; in vitro) sequences.

### Datasets

The yeast genome sequence (May 2006 build) and gene and chromosome annotations were obtained from SGD [63]. Yeast transcription start sites were compiled from [64]–[66]: for each gene, the transcription start site was taken as that with the most sequence reads from [64],[65], or from [66] when no sequencing data was available. Functional transcription factor DNA binding sites in yeast, defined as sites that are bound by their cognate transcription factor were obtained from [47],[67]. TATA elements in yeast were obtained from [53]. Functional annotations for yeast genes were downloaded from Gene Ontology [68]. Yeast genes bound by chromatin remodeling factors were obtained from [54]. Measurements of protein expression variability, referred to here as transcriptional noise, were obtained from [49]. Histone turnover rates at yeast promoters were obtained from [55]. Nucleosome-bound DNA sequences were obtained from: yeast [2],[5],[9],[10], worm [17], chicken [15]. Microarray-based nucleosome maps of yeast (3 maps) and human (1 map) were obtained from [5],[9],[10],[24].

### Computing the Nucleosome Fold Depletion of a Set of Genomic Regions

The nucleosome fold depletion over a set of genomic regions of interest is defined as the ratio between their expected and actual nucleosome coverage. The expected coverage is equal to the average number of nucleosomes that cover a basepair in the genome, computed by dividing the total number of basepairs covered by our 378,686 nucleosome reads, with the total number of basepairs in the genome that are not in the ribosomal DNA locus or in repetitive regions. The actual nucleosome coverage over a set of genomic regions is computed as above, but only across the basepairs in the given set of genomic regions. In our data, the expected coverage is 5.27. Thus, for example, a set of genomic elements whose actual average coverage per basepair is 0.1, is depleted by 5.27/0.1, or 52.7-fold.

### Defining Boundary Elements from Sequence

We use two sequence definitions for boundary elements. The first is based on single homopolymeric tracts of Poly-A or Poly-T (Poly(dA:dT) elements), and the second on clusters of short Poly(dA:dT) elements. For the definition based on a single Poly(dA:dT) element, we iterate over allowed values *k* = 0,1,2,…,20, for the number of mismatches relative to the Poly(dA:dT) tract. For each *k*, we then identify all maximal Poly(dA:dT) tracts in the genome with exactly *k* mismatches, where the mismatch cannot occur at the first or last basepair of the element. By maximal elements, we mean that if a Poly(dA:dT) element with exactly *k* mismatches is fully contained within a longer Poly(dA:dT) element with exactly *k* mismatches, then only the longer element is considered. For the definition based on clusters of short Poly(dA:dT) elements, we first define short Poly(dA:dT) elements as all Poly(dA:dT) elements with zero mismatches whose size is at least 5 bp. For each allowed value in the range *k* = 0,1,2,…,20, representing the number of mismatches, we then identify maximal clusters of the above short Poly(dA:dT) tracts with exactly *k* mismatches. As with single Poly(dA:dT) elements, mismatches cannot occur at the first or last basepair of each cluster and maximal elements are defined similarly. Note that in the definition based on Poly(dA:dT) clusters, the resulting boundaries may contain Poly(dA:dT) elements that alternate between strands (e.g., AAAAATTTTTT).

### Grouping Boundary Elements by Their Strength

For various analyses, we partitioned boundaries into distinct groups based on their nucleosome fold depletion, which we refer to as their strength. To this end, we first compute the nucleosome fold depletion (strength) over the set of boundaries with exactly *k* mismatches and whose length is at least *n*, for *k* = 0,1,2,…,20 and all values of *n* for which elements of that size exist. This computation is performed separately for each of the two boundary definitions above (single Poly(dA:dT) elements and clusters of Poly(dA:dT) elements). For a given requested partition of boundaries into strength groups, we then assign each set of boundaries with strength *s* to the strongest group among the groups whose strength is below *s*. Throughput this paper, we partitioned boundaries into groups of strength 2, 5, 10, and 20. Thus, for example, a set of boundaries whose strength is 30 will be assigned to the boundary group of strength 20. In cases of overlap in the genomic coordinates of boundary elements assigned to the same group, we take only the boundary with the smaller number of mismatches; and if the number of mismatches of the overlapping boundaries is the same, we take only the longer boundary. Finally, since the actual fold depletion of a boundary group by this procedure may differ from its original requested fold depletion, we compute and use the actual fold depletion of the boundary group for the various graphs that show plots as a function of boundary strength.

### Grouping Promoters by TATA Elements and Nucleosome Coverage over Factor Binding Sites

For the analyses of Figure 11, we grouped promoters into four classes, based on the presence of TATA elements and on whether or not their binding sites are covered by nucleosomes or are nucleosome-depleted. TATA boxes are taken from [53]. We classify a promoter as having sites that are covered by nucleosomes if at least 80% of the total basepairs of its binding sites are covered by at least one nucleosome read from our data. We classify a promoter as having sites that are nucleosome-depleted if at most 20% of the total basepairs of its binding sites are covered by at least one nucleosome read. Binding sites are taken from [47],[67].

### Sequence-Based Model for Nucleosome Positioning

We represent nucleosome sequence preferences using a probabilistic model that assigns a score to every 147 basepair (nucleosome-length) sequence. As discussed above, our model consists of two main components, each of which was separately and previously explored by published models. The first component, *P_N_*, represents the distribution over dinucleotides at each position along the nucleosome length, and thus captures the periodic signal of dinucleotides along the nucleosome. The second component, *P_L_*, represents the position-independent distribution over 5-mers at linker regions compared to nucleosomal DNA, and thus captures sequences that are generally favored or disfavored by nucleosomes regardless of their detailed position within the nucleosome. We chose to represent this component using 5-mers, since this is the highest order k-mer for which our data has sufficient statistics to robustly estimate each of the associated parameters, and the k-mer order that results in the highest AUC performance in a cross validation scheme. The final score that our model assigns to a 147 bp sequence *S* is then given by the log-ratio of these two model components:
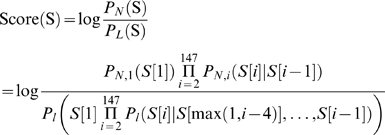
(1)where *P_N_*,*_i_* is the *i*th component of the dinucleotide model component and represents the conditional probability distribution over nucleotides at position *i* given the nucleotide that appeared at position (*i*−1), and *P_l_* is the position-independent component of the second component of our model (*P_L_*). Note that *P_N_*_,*1*_ is represented by a mononucleotide model over the nucleotide at the first position.

We now describe in detail how each of the two components of our model is derived. To compute the position-specific dinucleotide component of our model, *P_N_*, we start with a collection of nucleosome-bound sequences, and estimate *P_N_* from the 23,076 nucleosome sequence reads of length 146–148. We restricted ourselves to this length range of nucleosomes, since the border of the nucleosome is the most likely cut site for the nuclease and thus these nucleosome reads are likely to be mapped with the highest accuracy. Indeed, these nucleosome reads exhibit clear periodicities of dinucleotides along the nucleosome length, similar to those reported previously [2],[15] (Figure S2). For the estimation, we first align all sequences about their center, where each sequence is added twice to the alignment, once in its original form and once in its reverse complement form, to account for the 2-fold symmetry in the nucleosome structure [69]. Sequences of even length are treated as two sequences, each with a weight of 0.5, once in a configuration that has one more base at the left side of the alignment, and once in a configuration that has one more base to the right of the alignment. This accounts for the uncertainty we have in the positioning of the even length sequences relative to the center. With each position *i*, we then associate a dinucleotide distribution, *P_N_*_,*i*_, which we estimate from the combined dinucleotide counts at alignment positions [*i*−2, *i*−1], [*i*−1, *i*], and [*i*, *i*+1] (the two end positions of the nucleosome are averaged with less positions). Combining the dinucleotides at the two neighboring positions smoothes the resulting dinucleotide distribution at each position with a 3 basepair moving average, and is motivated by the experimental evidence that small ±1 basepair changes in spacing of key nucleosome DNA sequence motifs can occur with relatively small cost to the free energy of histone–DNA interactions [70]. To remove sequence composition biases from this component, we normalize the distribution, by dividing the final probability of every dinucleotide at each position by the probability of that dinucleotide across all positions, and finally normalize the resulting weights to a probability distribution. We used this estimation procedure in the 127 central positions of the nucleosome, and we force a uniform distribution over the 10 remaining positions at each end of the nucleosome profile. This was done to avoid biases in nucleotide distributions that may arise from the sequence specificity of the micrococcal nuclease used to isolate the nucleosome, since this way we do not include statistics that are taken from the cut site of the nuclease. Note that our above construction produces a reverse complement symmetric distribution, i.e., the probability of a sequence and its reverse complement are equal.

The position-independent component of our model, *P_L_*, whose purpose is to represent sequences that are generally favored or disfavored regardless of their position within the nucleosome, assigns a score to each 147 bp sequence, as the product of a position-independent Markov model, *P_l_*, of order 4. Thus, *P_l_* defines a probability distribution over every one of the 1024 possible 5-mers. We chose to model the distribution over 5-mers, since this is the highest order in which our data still provides sufficient statistics to robustly estimate the value of each of the 1024 parameters. Given a collection of nucleosome-bound sequences, we set the weight of each 5-mer to the ratio between the frequency of that 5-mer in the linkers, and the frequency of that 5-mer in the nucleosome-bound sequences, where this ratio is then scaled to be a probability by dividing it by the sum of ratios across all 5-mers. As linkers, we take all 8022 contiguous non-repetitive regions of length 50–500 bp that are not covered by any nucleosome from the input collection. All 344,976 nucleosome-bound sequences of length greater than 146 are taken as the set of nucleosomes, and statistics are collected only from their central 127 bp to avoid alignment issues whereby the outermost regions of any given nucleosome may in fact be linkers. From the above linker DNAs, we ignored the statistics of the 5 basepairs at the end of each linker, to avoid biases that may be introduced from the sequence specificity of the micrococcal nuclease used in our experiments to isolate nucleosomes. Thus, this Markov model, *P_l_*, includes contributions from both sequences that are disfavored by nucleosomes and sequences that are favored by nucleosomes, since it models the distribution over all 5-mers, with the disfavored sequences having a relatively high probability and the favored sequences having a relatively low probability.

We note, that although we discuss each of the two model components separately, these components are in fact not independent, since each component captures some aspects of the other component. For example, the position-independent component, *P_L_*, may capture position-specific dependencies between nucleotides separated by four basepairs, and such dependencies are part of the position-specific component, and vice versa for the periodic *P_N_* component.

### Thermodynamic Model for Predicting Nucleosome Positions Genome-Wide

The above probabilistic model assigns a nucleosome formation score to each sequence of (nucleosome-length) 147 bp. We then use the scores of this model to compute the genome-wide distribution over nucleosome positions, taking into account steric hindrance constraints between neighboring nucleosomes. To this end, we take the partition function to be the space of all *legal configurations* of nucleosomes on a sequence ***S***, where a legal configuration specifies a set of 147 bp nucleosomes and a start position for each of these nucleosomes on ***S***, such that no two nucleosomes overlap. A legal configuration thus respects a simple approximation of the detailed linker length-dependent steric hindrance constraints between nucleosomes. We score a sequence *S* for its apparent nucleosome binding affinity using the above formula for *Score*(*S*). For each sequence ***S*** and legal configuration *c* with *k* nucleosomes positioned at *c* [1],…,*c*[*k*], we assign a statistical weight *W_c_*[***S***] defined as:
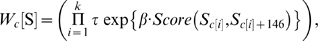
where *τ* represents an apparent nucleosome concentration, and *β* is an apparent inverse temperature parameter. Our default parameter settings are *τ* = 1 and *β* = 0.5. In accord with the Boltzmann distribution and under the assumption of thermodynamic equilibrium, it follows that the probability of every configuration is then given by:
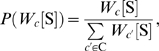
where *c′* goes over the space of all legal configurations ***C***. A dynamic programming method [2],[71] can efficiently compute the probability of placing a nucleosome that starts at each basepair in the genome. The underlying idea is that the probability of placing a nucleosome starting at a particular basepair *i* is equal to the sum of the statistical weights of all configurations in which a nucleosome starts at position *i*, divided by the sum of the statistical weights of all legal configurations. Both of these sums can be computed efficiently in three steps. The first is a *forward* step, in which we compute a set of variables *F_1_*,*…F_N_*, where *F_i_* represents the sum of the statistical weight of all legal configurations of the sub-sequence *S*_1_,…*S_i_*, as follows:
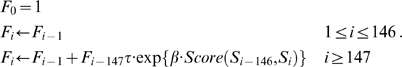



The second step is a *reverse step*, in which we compute a set of variables *R*_1_,…*R_N_*, where *R_i_* represents the statistical weight of all legal configurations of the sub-sequence *S_i_*,…*S_N_*, as follows:
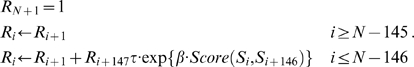



In the final step, we can directly compute the probability, *P*(*i*), of placing a nucleosome that starts at each basepair *i* of *S*, where *i*≤*N*−146, as follows:
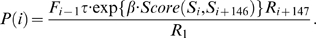



The probability that a basepair *i* in *S* is covered by any nucleosome, referred hereto after as the average nucleosome occupancy predicted by our model, is the sum of the probabilities of starting a nucleosome at any of the positions from *i*−146 to *i*, i.e., 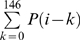.

## Supporting Information

Figure S1 Our map shows significant correspondence with microarray-based nucleosome maps. (A) Shown is the fraction of our nucleosome reads (blue solid line; *y*-axis) whose center is within a particular distance from the center of at least one nucleosome from the nucleosome calls from [1]. For this plot, we only considered nucleosome reads from our data that are contained in regions that were mapped by probes from the microarray of [1], and we filtered our nucleosome reads to contain only unique nucleosome centers, by representing multiple nucleosome reads that have the same center as a single nucleosome. To assess the significance of the correspondence, we permuted the locations of our unique set of nucleosomes within the regions covered by the microarray of [1], and repeated this same plot for the permuted nucleosome set (dotted blue line; *y*-axis). (B) Same as (A), but where the fraction of nucleosomes shown is the reverse, i.e., the fraction of nucleosome calls from [1] (red solid line; *y*-axis) whose center is within a particular distance from the center of at least one nucleosome from our nucleosome reads. (C,D) Same as (A,B), for a comparison against the microarray nucleosome map of [2]. (E,F) Same as (A,B), for a comparison against the microarray nucleosome map of [3]. (G,H) Same as (A,B), for a comparison against the sequence-based nucleosome map of [4]. (I,J) Same as (A,B), for a comparison against the sequence-based map of H2A.Z nucleosomes from [5]. (K,L) Same as (A,B), for a comparison against 99 nucleosomes mapped in the literature, and compiled in [6].
(0.53 MB TIF)

Figure S2 Periodicity of dinucleotides along the nucleosome length. Frequencies of all 16 dinucleotides at each position of our center-aligned nucleosome-bound sequences with length 146–148.
(0.34 MB TIF)

Figure S3 Comparison of different models for nucleosome positioning. (A) Evaluation of the abilities of various models to separate linkers from nucleosomal DNA. For every model tested, shown is the fraction of all measured nucleosomes that the model correctly classifies as nucleosomes (*y*-axis; true positive rate) against the fraction of all measured linkers that the model incorrectly classifies as nucleosomes (*x*-axis; false positive rate), for each possible threshold on the minimum score above which the model classifies a region as nucleosomal. For every model tested, the result is a standard receiver operating characteristic (ROC) curve, whose area under the curve (AUC; shown in inset) is a quantitative measure of the quality of the predictions, with the value 1 being perfect and 0.5 being random guessing. The score of each measured nucleosome (or linker) is the mean score that the model assigns in the region that is 20 bp from the center of the nucleosome (linker). For our model, scores are assigned once using a cross validation scheme (orange line; annotated “Our model CV”), in which every nucleosome or linker on a given chromosome is assigned a score using a model that was trained from the data of all other chromosomes, and once using all the data for training (blue line; annotated “Our model”). For the other five published models being compared, scores were taken from the models trained by the authors. For the model of [7] (purple line; annotated “Peckham (07)”), which was designed to assign raw scores to every 50 basepairs in the genome, the score of each 147 bp nucleosome was taken to be the average score of all 50 bp regions contained within the 147 bp. For the model of [1] (cyan line; annotated “Lee (07)”), scores for 147 bp regions were downloaded from the authors' website. For the model of [8] (green line; annotated “Yuan (08)”), scores were computed by applying code obtained from Dr. Yuan to every 147 bp region. For the model of [9] (brown line; annotated “Ioshikes (06)”), scores were computed by applying code obtained from Dr. Yuan to every 147 bp region. For the model of [6] (red line; annotated “Segal (06)”) scores were downloaded from the authors' website. For all of these comparisons, linkers are taken as contiguous non-repetitive regions of lengths 50–500 bp that are not covered by any nucleosome in our data. Results are shown for separating these linkers from all of the nucleosomes in our data. (B) Same as (A), for separating linkers and nucleosomes that agree between two genome-wide microarray-based nucleosome maps in yeast1,3. To this end, we took the reported nucleosome positions from [1] that were obtained by applying an HMM to the hybridization signals, and the reported nucleosome positions of [3]. As linkers, we took contiguous regions of lengths 50–500 bp that are nucleosome-free in both maps. As nucleosomes, we took nucleosomes whose center was within 20 bp of the center of a reported nucleosome position in the other dataset. Note that the model of [1] was learned from one of the two microarrays (from [1]) on which the evaluation is shown.
(0.27 MB TIF)

Figure S4 Large nucleosome depletion over Poly(dA:dT) elements. Graphs showing the nucleosome fold depletion over Poly(dA:dT) elements as in Figure 2D, but for all possible number of mismatches 0,1,2,…,20. (A) The number of elements in each of the points shown in every graph from (panel c). (B) The number of elements in each of the points shown in every graph from (panel d). (C) Shown is the combined nucleosome fold depletion over all homopolymeric tracts of A's or T's (Poly(dA:dT) elements) of length *k*, for *k* = 5,6,7,…, and for Poly(dA:dT) elements with exactly 0,1,2,…,20 base substitutions (mismatches). Each graph is trimmed at a length *K* in which there are less than 10 elements, and the fold depletion at this final point is computed over all elements whose length is at least *K*. The combined fold depletion of a set of genomic elements (*y*-axis) is the ratio between their expected and observed nucleosome coverage, where the expected coverage is the average coverage of any basepair according to our data, and the observed coverage is the average coverage of a basepair from the set (see Methods). (D) As in (C), but for clusters of perfect Poly(dA:dT) elements, where each element is at least 5 bp, and where the total number of bases in the cluster that are not in perfect Poly(dA:dT) elements (mismatches) is exactly 0,1,2,…,20.
(0.64 MB TIF)

Figure S5 Number and genomic distribution of boundaries in the yeast genome. (A) Shown is the number of sequence boundaries in the yeast genome at various boundary strengths. The strength of a boundary is a measure of its level of nucleosome fold depletion and is defined using our data (see Methods). The graph displays the overall number of boundaries (orange) and the number of boundaries that intersect gene coding regions (green), promoter regions (blue), 5′ untranslated regions (5′ UTRs; red), and intergenic regions that are not promoters (brown). (B) Same as (A), but represented as the frequency of boundary per basepair across the entire genome (orange) and across the different types of genomic regions from (A).
(0.17 MB TIF)

Figure S6 Strong depletion in vitro over a Poly(dA:dT) element in sheep. Shown are intensity measurements (*y*-axis) from [10], corresponding to nucleosome occupancies at 1743 positions from a ∼10 kb region around the β-Lactoglobulin locus of sheep, after in vitro nucleosome reconstitution on this region. Positions are given relative to the transcription start site of the gene. The only Poly-T(13) element in the region is indicated, along with the sequence context in which it is embedded. Note the strong nucleosome depletion over this element.
(0.33 MB TIF)

Figure S7 Genes with high- and low-noise promoter architectures exhibit many (and different) functional enrichments. For each of the four gene groups from (Figure 11A), shown is their functional enrichment for genes with particular functional categories from GO11, transcription factor binding sites12,13, targets of chromatin remodeling complexes3,14, and essential genes15. The two extreme promoter types from Figure 11A (type I: first row, genes with TATA elements and nucleosome-covered factor sites; type II: fourth row, genes without TATA elements and with nucleosome-depleted factor sites) show many significant enrichments, in contrast to the two other promoter types. The *p*-value of a hypergeometric test is given for each category, along with the number of genes from the group annotated as belonging to the category (first number in parentheses), the number of genes from the group (second number), the number of genes annotated as belonging to the category (third number), and the total number of genes that were both part of our four groups and were annotated as belonging or not to the category (fourth number). We report only *p*-values less than 10^−5^.
(0.40 MB TIF)
