# Supplementary figures and images for: Distinct Modes of Regulation by Chromatin Encoded through Nucleosome Positioning Signals
Source: PLoS Comput Biol. 2008 Nov 7;4(11):e1000216. doi: 10.1371/journal.pcbi.1000216 (PMC2570626; doi:10.1371/journal.pcbi.1000216)

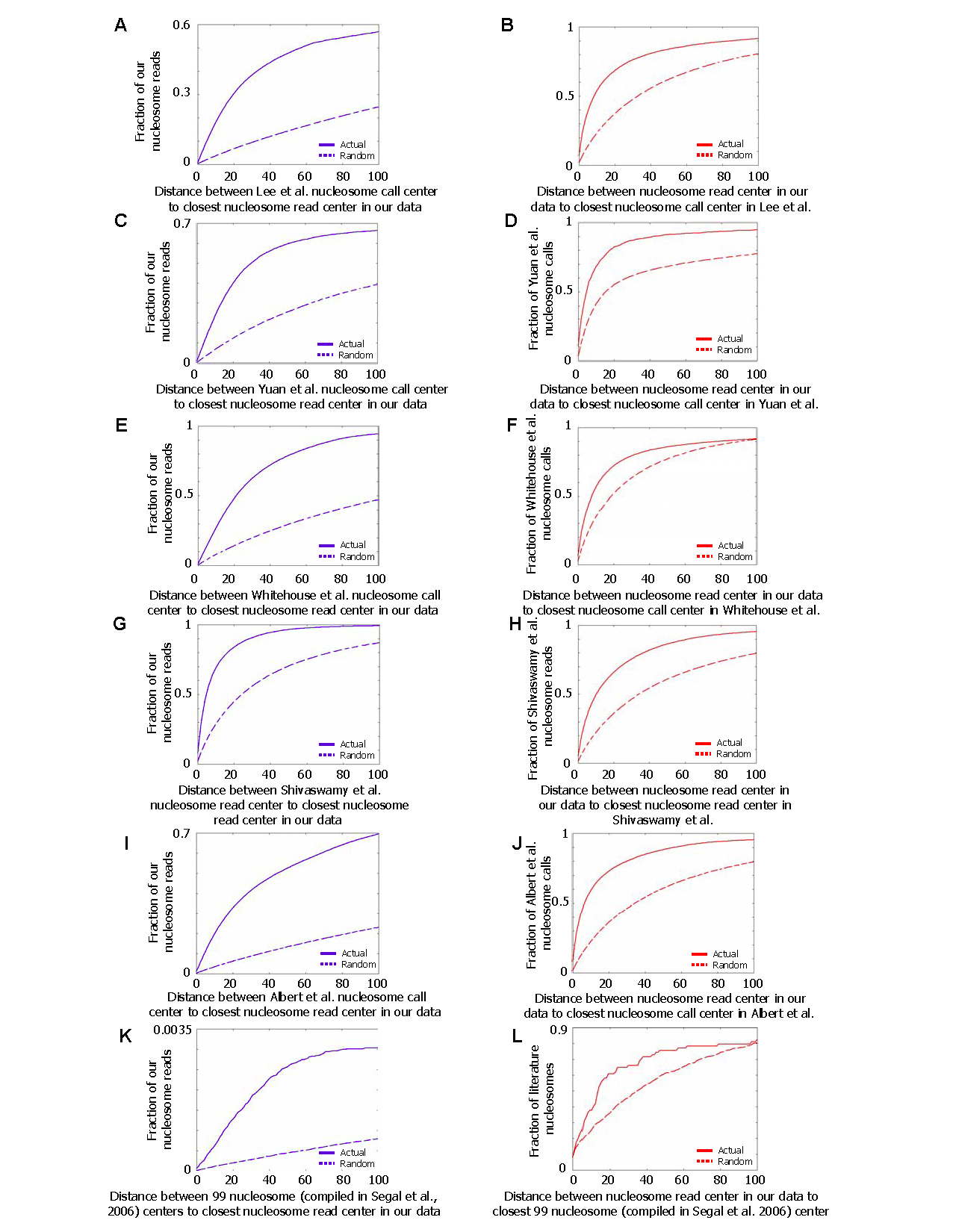

Supplement: Figure S1 — Our map shows significant correspondence with microarray-based nucleosome maps. (A) Shown is the fraction of our nucleosome reads (blue solid line; y-axis) whose center is within a particular distance from the center of at least one nucleosome from the nucleosome calls from [1]. For this plot, we only considered nucleosome reads from our data that are contained in regions that were mapped by probes from the microarray of [1], and we filtered our nucleosome reads to contain only unique nucleosome centers, by representing multiple nucleosome reads that have the same center as a single nucleosome. To assess the significance of the correspondence, we permuted the locations of our unique set of nucleosomes within the regions covered by the microarray of [1], and repeated this same plot for the permuted nucleosome set (dotted blue line; y-axis). (B) Same as (A), but where the fraction of nucleosomes shown is the reverse, i.e., the fraction of nucleosome calls from [1] (red solid line; y-axis) whose center is within a particular distance from the center of at least one nucleosome from our nucleosome reads. (C,D) Same as (A,B), for a comparison against the microarray nucleosome map of [2]. (E,F) Same as (A,B), for a comparison against the microarray nucleosome map of [3]. (G,H) Same as (A,B), for a comparison against the sequence-based nucleosome map of [4]. (I,J) Same as (A,B), for a comparison against the sequence-based map of H2A.Z nucleosomes from [5]. (K,L) Same as (A,B), for a comparison against 99 nucleosomes mapped in the literature, and compiled in [6]. (0.53 MB TIF) [file pcbi.1000216.s001.tif]

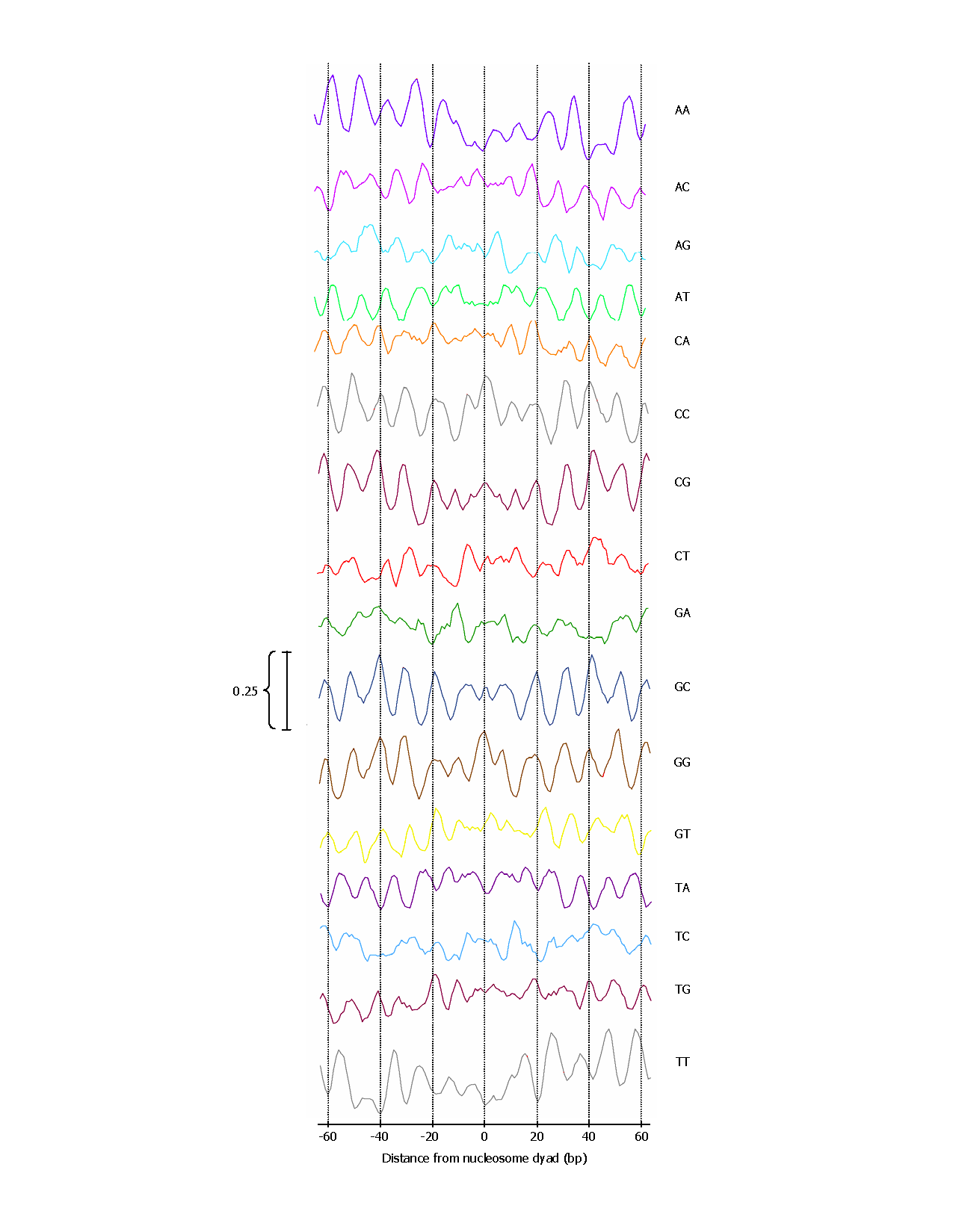

Supplement: Figure S2 — Periodicity of dinucleotides along the nucleosome length. Frequencies of all 16 dinucleotides at each position of our center-aligned nucleosome-bound sequences with length 146–148. (0.34 MB TIF) [file pcbi.1000216.s002.tif]

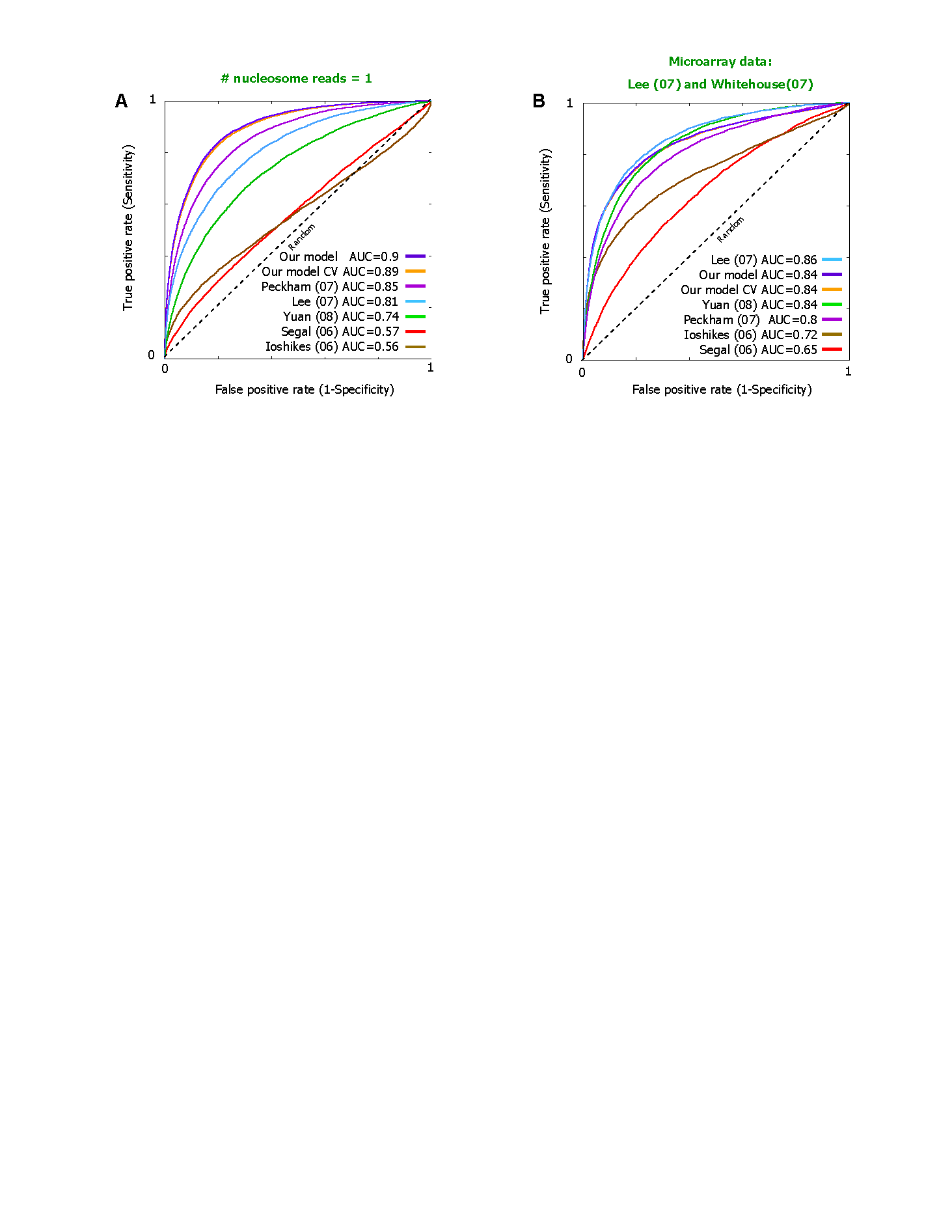

Supplement: Figure S3 — Comparison of different models for nucleosome positioning. (A) Evaluation of the abilities of various models to separate linkers from nucleosomal DNA. For every model tested, shown is the fraction of all measured nucleosomes that the model correctly classifies as nucleosomes (y-axis; true positive rate) against the fraction of all measured linkers that the model incorrectly classifies as nucleosomes (x-axis; false positive rate), for each possible threshold on the minimum score above which the model classifies a region as nucleosomal. For every model tested, the result is a standard receiver operating characteristic (ROC) curve, whose area under the curve (AUC; shown in inset) is a quantitative measure of the quality of the predictions, with the value 1 being perfect and 0.5 being random guessing. The score of each measured nucleosome (or linker) is the mean score that the model assigns in the region that is 20 bp from the center of the nucleosome (linker). For our model, scores are assigned once using a cross validation scheme (orange line; annotated “Our model CV”), in which every nucleosome or linker on a given chromosome is assigned a score using a model that was trained from the data of all other chromosomes, and once using all the data for training (blue line; annotated “Our model”). For the other five published models being compared, scores were taken from the models trained by the authors. For the model of [7] (purple line; annotated “Peckham (07)”), which was designed to assign raw scores to every 50 basepairs in the genome, the score of each 147 bp nucleosome was taken to be the average score of all 50 bp regions contained within the 147 bp. For the model of [1] (cyan line; annotated “Lee (07)”), scores for 147 bp regions were downloaded from the authors' website. For the model of [8] (green line; annotated “Yuan (08)”), scores were computed by applying code obtained from Dr. Yuan to every 147 bp region. For the model of [9] (brown line; annotated “Ioshike [file pcbi.1000216.s003.tif]

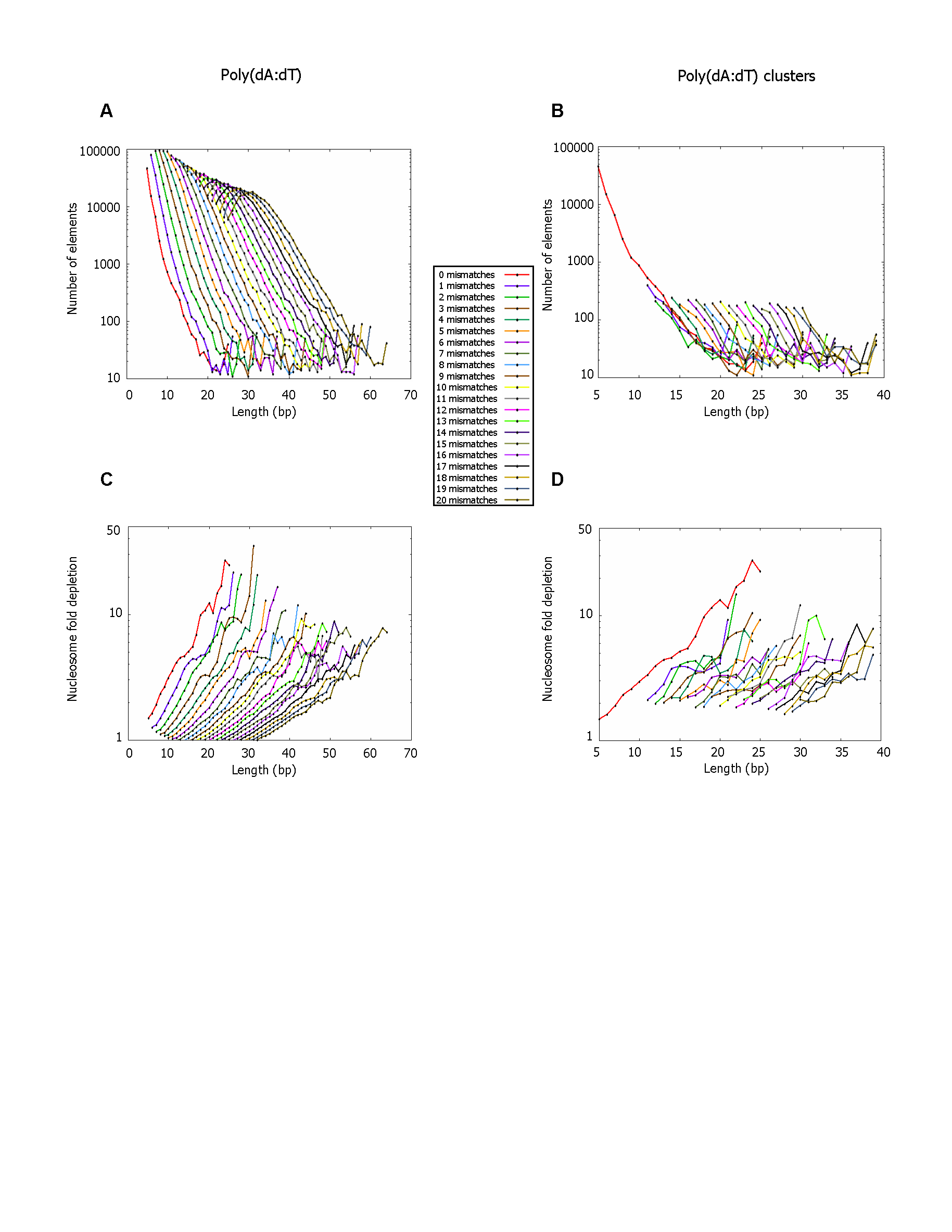

Supplement: Figure S4 — Large nucleosome depletion over Poly(dA:dT) elements. Graphs showing the nucleosome fold depletion over Poly(dA:dT) elements as in Figure 2D, but for all possible number of mismatches 0,1,2,…,20. (A) The number of elements in each of the points shown in every graph from (panel c). (B) The number of elements in each of the points shown in every graph from (panel d). (C) Shown is the combined nucleosome fold depletion over all homopolymeric tracts of A's or T's (Poly(dA:dT) elements) of length k, for k = 5,6,7,…, and for Poly(dA:dT) elements with exactly 0,1,2,…,20 base substitutions (mismatches). Each graph is trimmed at a length K in which there are less than 10 elements, and the fold depletion at this final point is computed over all elements whose length is at least K. The combined fold depletion of a set of genomic elements (y-axis) is the ratio between their expected and observed nucleosome coverage, where the expected coverage is the average coverage of any basepair according to our data, and the observed coverage is the average coverage of a basepair from the set (see Methods). (D) As in (C), but for clusters of perfect Poly(dA:dT) elements, where each element is at least 5 bp, and where the total number of bases in the cluster that are not in perfect Poly(dA:dT) elements (mismatches) is exactly 0,1,2,…,20. (0.64 MB TIF) [file pcbi.1000216.s004.tif]

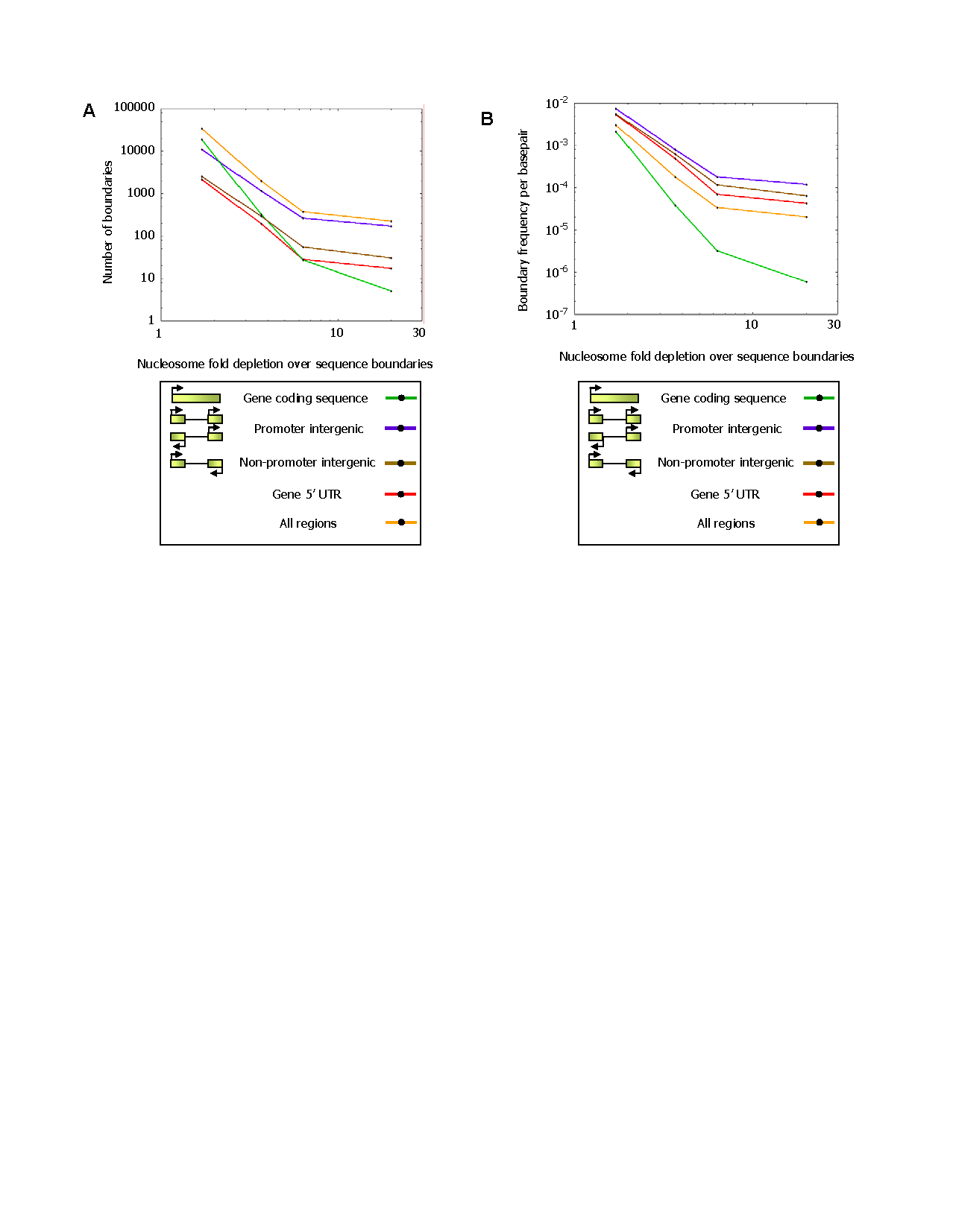

Supplement: Figure S5 — Number and genomic distribution of boundaries in the yeast genome. (A) Shown is the number of sequence boundaries in the yeast genome at various boundary strengths. The strength of a boundary is a measure of its level of nucleosome fold depletion and is defined using our data (see Methods). The graph displays the overall number of boundaries (orange) and the number of boundaries that intersect gene coding regions (green), promoter regions (blue), 5′ untranslated regions (5′ UTRs; red), and intergenic regions that are not promoters (brown). (B) Same as (A), but represented as the frequency of boundary per basepair across the entire genome (orange) and across the different types of genomic regions from (A). (0.17 MB TIF) [file pcbi.1000216.s005.tif]

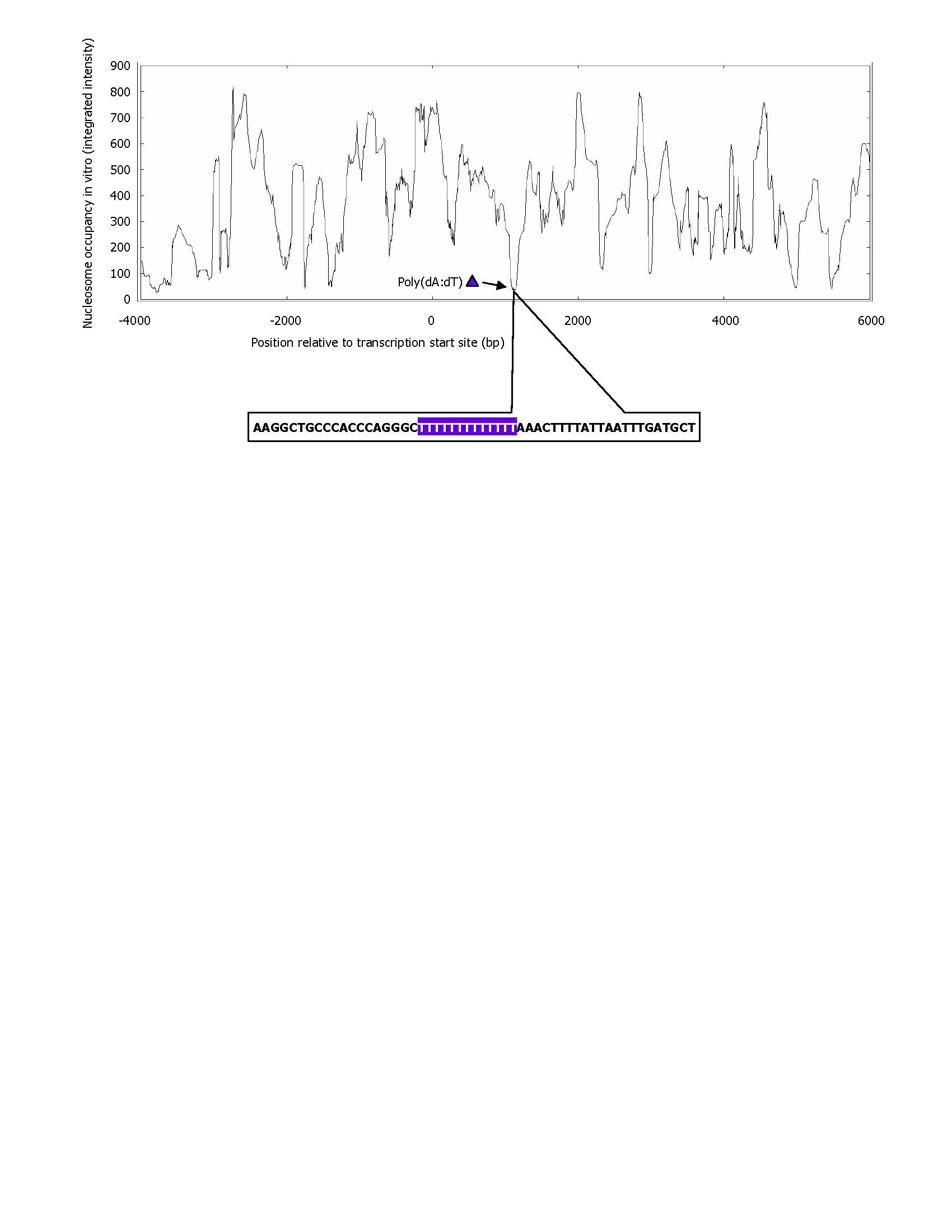

Supplement: Figure S6 — Strong depletion in vitro over a Poly(dA:dT) element in sheep. Shown are intensity measurements (y-axis) from [10], corresponding to nucleosome occupancies at 1743 positions from a ∼10 kb region around the β-Lactoglobulin locus of sheep, after in vitro nucleosome reconstitution on this region. Positions are given relative to the transcription start site of the gene. The only Poly-T(13) element in the region is indicated, along with the sequence context in which it is embedded. Note the strong nucleosome depletion over this element. (0.33 MB TIF) [file pcbi.1000216.s006.tif]

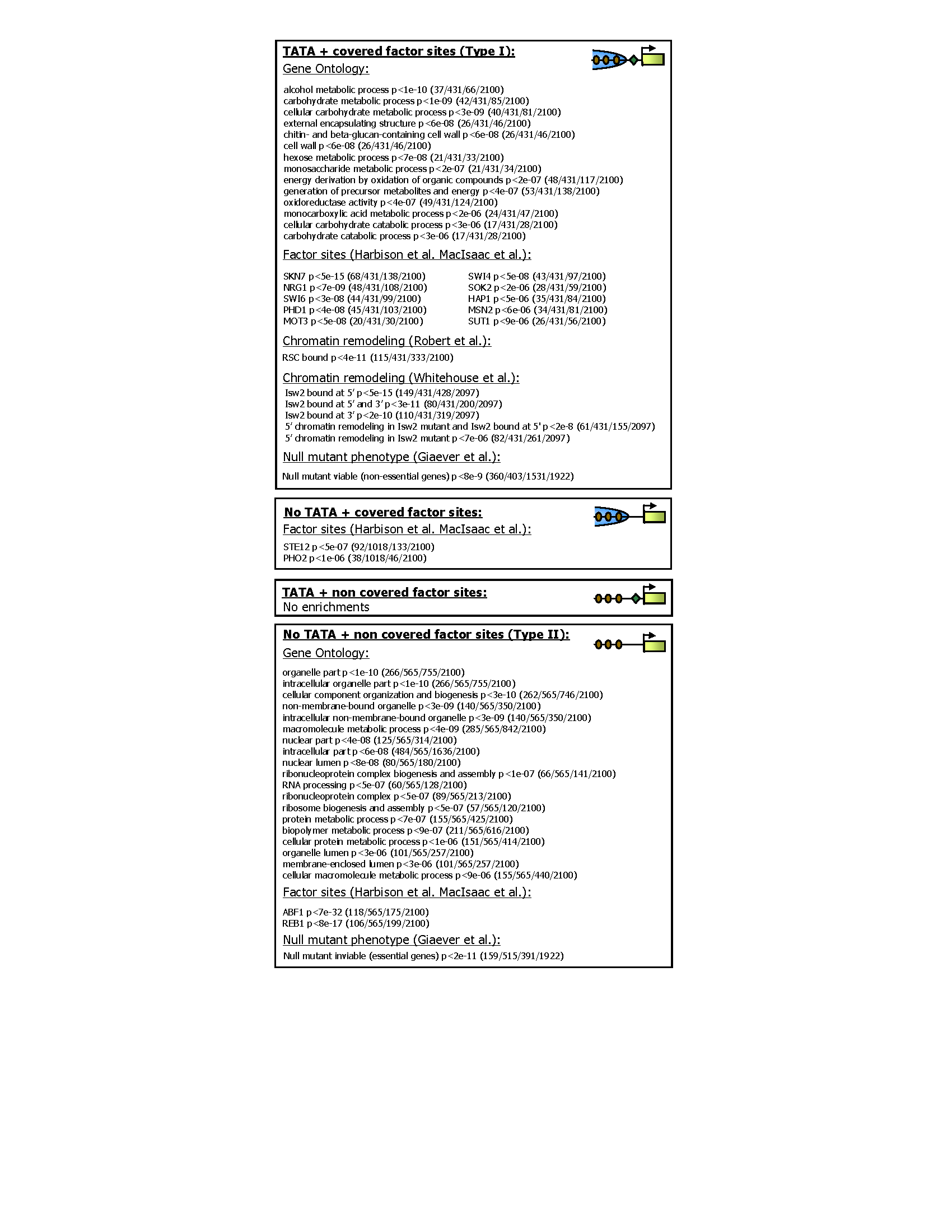

Supplement: Figure S7 — Genes with high- and low-noise promoter architectures exhibit many (and different) functional enrichments. For each of the four gene groups from (Figure 11A), shown is their functional enrichment for genes with particular functional categories from GO11, transcription factor binding sites12,13, targets of chromatin remodeling complexes3,14, and essential genes15. The two extreme promoter types from Figure 11A (type I: first row, genes with TATA elements and nucleosome-covered factor sites; type II: fourth row, genes without TATA elements and with nucleosome-depleted factor sites) show many significant enrichments, in contrast to the two other promoter types. The p-value of a hypergeometric test is given for each category, along with the number of genes from the group annotated as belonging to the category (first number in parentheses), the number of genes from the group (second number), the number of genes annotated as belonging to the category (third number), and the total number of genes that were both part of our four groups and were annotated as belonging or not to the category (fourth number). We report only p-values less than 10−5. (0.40 MB TIF) [file pcbi.1000216.s007.tif]
